# DNA methylation is involved in the regulation of pepper fruit ripening and interacts with phytohormones

**DOI:** 10.1093/jxb/eraa003

**Published:** 2020-01-07

**Authors:** Kai Xiao, Jie Chen, Qixiumei He, Yixin Wang, Huolin Shen, Liang Sun

**Affiliations:** 1 Department of Vegetable Science, College of Horticulture, China Agricultural University, Beijing, P.R. China; 2 Beijing Key Laboratory of Growth and Developmental Regulation for Protected Vegetable Crops, Department of Vegetable Science, College of Horticulture, China Agricultural University, Beijing, P.R. China; 3 Fondazione Edmund Mach, Italy

**Keywords:** Carotenoids, DNA methylation, fruit ripening, gene expression, pepper, plant hormone

## Abstract

There is growing evidence to suggest that epigenetic tags, especially DNA methylation, are critical regulators of fruit ripening. To examine whether this is the case in sweet pepper (*Capsicum annuum*) we conducted experiments at the transcriptional, epigenetic, and physiological levels. McrBC PCR, bisulfite sequencing, and real-time PCR demonstrated that DNA hypomethylation occurred in the upstream region of the transcription start site of some genes related to pepper ripening at the turning stage, which may be attributed to up-regulation of *CaDML2-like* and down-regulation of *CaMET1-like1*, *CaMET1-like2*, *CaCMT2-like*, and *CaCMT4-like*. Silencing of *CaMET1-like1* by virus-induced gene silencing led to DNA hypomethylation, increased content of soluble solids, and accumulation of carotenoids in the fruit, which was accompanied by changes in expression of genes involved in capsanthin/capsorubin biosynthesis, cell wall degradation, and phytohormone metabolism and signaling. Endogenous ABA increased during fruit ripening, whereas endogenous IAA showed an opposite trend. No ethylene signal was detected during ripening. DNA hypomethylation repressed the expression of auxin and gibberellin biosynthesis genes as well as cytokinin degradation genes, but induced the expression of ABA biosynthesis genes. In mature-green pericarp, exogenous ABA induced expression of *CaDML2-like* but repressed that of *CaCMT4-like*. IAA treatment promoted the transcription of *CaMET1-like1* and *CaCMT3-like*. Ethephon significantly up-regulated the expression of *CaDML2-like*. Treatment with GA_3_ and 6-BA showed indistinct effects on DNA methylation at the transcriptional level. On the basis of the results, a model is proposed that suggests a high likelihood of a role for DNA methylation in the regulation of ripening in the non-climacteric pepper fruit.

## Teaser

Down-regulation of *CaMET1-like1* in pepper leads to DNA hypomethylation and premature fruit ripening, and interactions between DNA methylation and phytohormones appear to fine-tune the ripening process.

## Introduction

The ripening of fleshy fruit involves an elaborate process that is regulated by many factors at multiple levels. There is growing evidence to suggest that epigenetic tags are a critical regulator of fruit ripening, especially DNA methylation (Zhong *et al.*, 2013; Liu *et al.*, 2015; Lang *et al.*, 2017; Cheng *et al.*, 2018).

DNA methylation mainly refers to the methyl at the 5´ position of cytosine (5mC) (Bender, 2004). Based on the neighboring nucleotides, it can be classified into symmetric (CG or CHG) and asymmetric (CHH) types (Deleris *et al.*, 2016). The formation of asymmetric DNA methylation is catalysed by CHROMOMETHYLASE 2 (CMT2) and DOMAIN REARRANGED METHYLTRANSFERASES (DRM1 and DRM2) through the RNA-directed DNA methylation (RdDM) pathway, whereas the symmetric type is catalysed by METHYLTRANSFERASE 1 (MET1) and CHROMOMETHYLASE 3 (CMT3) (Finnegan and Kovac, 2000; Lindroth *et al.*, 2001; Bender, 2004; Zhang *et al.*, 2010; Deleris *et al.*, 2016). DNA methylation can be passively lost after DNA replication in the absence of methyltransferase activity or it can be actively eliminated by the DNA demethylases REPRESSOR OF SILENCING 1 (ROS1), DEMETER (DME), DME-LIKE 2 (DML2), and DME-LIKE 3 (DML3), which remove the 5mC base and then fill the single-nucleotide gap with a non-methylated cytosine (Gong *et al.*, 2002; Hsieh *et al.*, 2009; Wu and Zhang, 2010; Liu *et al.*, 2015; Lang *et al.*, 2017).

DNA methylation has been demonstrated to regulate fruit ripening in tomato, strawberry, and sweet orange. A global decrease of DNA methylation, especially at the 5´- and 3´-regulatory regions of the genes, has been associated with tomato and strawberry fruit ripening, and artificially accelerating this process by application of the methyltransferase inhibitor 5‐azacytidine induces premature ripening (Zhong *et al.*, 2013; Cheng *et al.*, 2018). In contrast, inhibition of ripening-associated hypomethylation through knockdown of *SlDML2* disturbs the natural dynamic expression patterns of many tomato genes related to ripening and ultimately leads to retarded ripening (Liu *et al.*, 2015; Lang *et al.*, 2017). In the case of sweet orange, a global hypermethylation of DNA is found in the peel, which is critical for the ripening-associated process of de-greening (Huang *et al.*, 2019). Differentially expressed genes regulated by ripening-associated changes in DNA methylation are usually enriched in the processes of pigment biosynthesis (such as anthocyanin biosynthesis in strawberry and carotenoid biosynthesis in tomato), aromatic compound biosynthesis, cell wall degradation, and ripening-related phytohormone biosynthesis (such as ABA biosynthesis in strawberry and sweet orange, and ethylene biosynthesis in tomato) (Lang *et al.*, 2017; Cheng *et al.*, 2018; Huang *et al.*, 2019). The ripening-associated variation in DNA methylation is controlled by DNA methyltransferase and demethylase genes at the transcriptional level. In tomato, the ripening-associated DNA hypomethylation is attributed to both the down-regulation of DNA methyltransferase genes and the up-regulation of DNA demethylase genes (Cao *et al.*, 2014). On the other hand, in strawberry this epigenetic change is mainly caused by the down-regulated of genes involving in RdDM (Cheng *et al.*, 2018). In the peel of sweet orange, ripening-associated DNA hypermethylation is primarily caused by the down-regulation of DNA demethylase genes (Huang *et al.*, 2019).

In contrast with the recent evidence for the involvement of epigenetic tags, the role of phytohormones in fruit ripening is long established. Ethylene plays a critical role in triggering ripening in climacteric fruit, and suppression of its biosynthesis and/or blocking of its signal pathway severely delays the normal ripening process (Oeller *et al.*, 1991; Lanahan *et al.*, 1994; Li *et al.*, 2007; Lee *et al.*, 2012; Sekeli *et al.*, 2014). ABA is well accepted as an important positive regulator of ripening in both climacteric and non-climacteric fruit (Jia *et al.*, 2011; Sun et al., 2012*a*, 2012*b*; Ji *et al.*, 2014; Wang et al., 2013, 2017). The accumulation of ABA is associated with ripening in many fleshy fruits, such as tomato (Sun et al., 2012*a*, 2012*b*), cucumber (Wang *et al.*, 2013), watermelon (Wang *et al.*, 2017), grape (Sun *et al.*, 2010), and strawberry (Jia *et al.*, 2011). Artificially inhibiting ABA accumulation and/or blocking its perception results in retarded fruit ripening (Chai *et al.*, 2011; Jia *et al.*, 2011; Sun et al., 2012*a*, 2012*b*; Ji *et al.*, 2014). Auxin has been found to have contrasting functions in the regulation fruit ripening. In most cases, it inhibits ripening by antagonizing the effects of ethylene or ABA (Manning *et al.*, 1994; Davies *et al.*, 1997; Liu *et al.*, 2005; Böttcher *et al.*, 2011; Symons *et al.*, 2012; Ziliotto *et al.*, 2012; Hao *et al.*, 2015; Su *et al.*, 2015; Li et al., 2016, 2017; Liao *et al.*, 2018; Sravankumar *et al.*, 2018); however, in some cases, especially in climacteric fruits such as tomato, peach, and fig, auxin promotes ripening by inducing the expression of ethylene biosynthesis genes (Gillaspy *et al.*, 1993; Jones *et al.*, 2002; Owino *et al.*, 2006; Trainotti *et al.*, 2007). Gibberellin generally plays a repressive role in the regulation of fleshy fruit ripening through antagonizing the effects of ethylene (Dostal and Leopold, 1967; Biale, 1978; Martínez *et al.*, 1994; Martínez-Romero *et al.*, 2000; Alós *et al.*, 2006; Kader, 2008). Our knowledge about the role of cytokinin in the regulation of fleshy fruit ripening is very limited, although it has been reported to repress ripening in grape berries (Peppi and Fidelibus, 2008).

Pepper (*Capsicum annuum*) is a fruit that is consumed worldwide and it is sold at the mature and ripening stages. Ripening determines pepper fruit quality because, on one hand, it promotes the accumulation of pigments and other nutrients that increase the quality of the ripe fruit, and on the other hand, post-harvest ripening and coloration severely reduce fruit quality and shelf-life of mature-green peppers. To date, our understanding of pepper fruit ripening is very limited. Pepper is currently considered as a medium type between climacteric and non-climacteric fruits (Villavicencio *et al.*, 2001) since some varieties, especially hot peppers, show an ethylene burst during ripening (Villavicencio *et al.*, 1999; Hou *et al.*, 2018) whereas some varieties, such as sweet peppers, do not (Lurie and Ben-Yehoshua 1986; Pretel *et al.*, 1995; Watkins, 2002). The regulatory mechanism of pepper fruit ripening remains unclear, although a few studies have found that ethylene and ABA seem to promote ripening whilst auxin, gibberellin, and cytokinin are likely to play opposite roles (Deruère *et al.*, 1994; Hou *et al.*, 2018). Whether DNA methylation affects the regulation of ripening and interacts with phytohormones in pepper is even more unclear. In this study, we therefore examined the effects of DNA methylation on pepper fruit ripening and investigated its interactions with phytohormones. Our results demonstrated that the degree of DNA methylation in the fruit may be controlled by DNA methyltransferase and demethylase genes at the transcriptional level. DNA hypomethylation caused by silencing of *CaMET1-like1* led to premature ripening. DNA methylation interacted with phytohormones in a way that may fine-tune the ripening of the fruit.

## Materials and methods

### Plant material

The sweet pepper (*Capsicum annuum* L.) inbred line 16C391 was developed in our lab and bears non-climacteric fruit. Plants used for developmental studies were grown in a greenhouse under natural daylight at the Experimental Station of China Agricultural University in 2017 and 2018. Plants were supplied with adequate water and nutrients according to standard horticultural practice. Pericarps were collected at the following stages: immature-green (IM, 15 d post anthesis, DPA), mature-green (MG, 38 DPA), breaker (B, 44 DPA), turning (T, 50 DPA), and red-ripening (R, 55 DPA). They were immediately frozen with liquid nitrogen and stored at –80 °C.

### McrBC-PCR analysis

McrBC-PCR was conducted to test the DNA methylation levels in the upstream regions of the transcriptional start site (UROT) of 12 ripening-related genes, namely *CaPSY1* (*Capana04g002519*, encoding phytoene synthase), *CaPDS* (*Capana03g000054*, encoding phytoene desaturase), *CaCCS* (*Capana06g000615*, encoding capsanthin-capsorubin synthase), *CaPG* (*Capana10g002229*, encoding polygalacturonase), *CaPME2.2* (*Capana00g004152*, encoding a pectin methylesterase), *CaCEL1* (*Capana01g000166*, encoding an endo-1,4-beta-glucanase), *CaCEL2* (*Capana08g002622*, encoding an endo-1,4-beta-glucanase), *CaNCED1* (*Capana00g003114*, encoding a 9-*cis*-epoxycarotenoid dioxygenase, a key enzyme in ABA biosynthesis), *CaCYP707A1* (*Capana04g001637*, encoding an 8′‐hydroxylase, a key enzyme in ABA catabolism), *CaRIN* (*Capana11g002005*, encoding a MADS-box transcription factor), *CaCNR* (*Capana02g001917*, encoding a MADS-box transcription factor), and *CaNOR* (*Capana07g002220*, encoding a NAC domain transcription factor). Genomic DNA was extracted using the CTAB method according to Healey *et al.* (2014) from ~3 g pericarp tissue collected at the IM and T stages. McrBC digestion was performed with 1 µg of genomic DNA using a McrBC kit (NEB Beijing, China) following the manufacturer’s instructions. The digestion system without GTP was used as a negative control. The tested regions (~800–1000 bp) with relatively higher GC level were selected from the 2-kb regions upstream the putative transcriptional start sites (TSSs) (Supplementary Fig. S1 at *JXB* online). The GC level was calculated using the MethPrimer software (Li and Dahiya, 2002). Primers were designed using Primer5 and are listed in Supplementary Table S1. PCR was performed with 50 ng of DNA as the template and the products were analysed using 1.5% agarose gel electrophoresis.

### Bisulfite sequencing

Bisulfite sequencing was performed to further confirm the DNA methylation level. DNA bisulfite conversion was conducted with 1 µg of genomic DNA using the a DNA Bisulfite Conversion Kit (Tiangen, China) following the manufacturer’s instructions. Methylation-specific PCR was set up with ~100 ng converted or non-converted genomic DNA as the template using a Methylation-specific PCR Kit (Tiangen, China) according to the user’s manual. Primers for the methylation-specific PCR were designed within the McrBC-PCR tested regions and are listed in Supplementary Table S2. The length of the amplification fragments ranged from 150–300 bp (Supplementary Fig. S1). Since every cytosine can be potentially methylated, to avoid any sequence selection bias during the PCR, the ‘C’ and ‘G’ nucleotides were replaced by ‘Y’ and ‘R’ in the forward and reverse primers, respectively. Ten single colonies for each PCR fragment were sequenced. The methylation ratio for each C-G site was calculated by dividing the number of non-changed nucleotides by the sequencing depth.

### Identification, phylogenetic analysis, and prediction of conserved functional regions of DNA methyltransferase and demethylase genes

BLAST searches were performed with the Arabidopsis and tomato orthologs as queries against the pepper genome in the NCBI (https://www.ncbi.nlm.nih.gov/), SOL (https://www.solgenomics.net), and The Pepper Genome (http://peppersequence.genomics.cn/page/species/index.jsp) databases. A Neighbor-joining phylogenetic tree was constructed using the ClustalX2.0.12 and MEGA4.0.2 software (bootstrap =1000 replicates). Conserved functional regions were predicted using a MOTIF Search (https://www.genome.jp/tools/motif/) with the default settings.

### Quantitative real-time PCR analysis

Total RNA extraction, first strand cDNA synthesis, and real-time PCR were conducted according to Sun *et al.* (2011). *UBI-3* (*Capana06g002873*) was selected as the internal control (Wan *et al.*, 2011) and relative expression values were calculated using the 2^–∆∆*C*T^ method (Livak and Schmittgen, 2001). For each gene, three biological replicates were performed, and for each biological replicate at least three technical replicates were performed. Primers used in this analysis are listed in Supplementary Table S3.

### Subcellular localization of *CaMET1-like1*, *CaCMT3-like*, and *CaDML2-like*

The coding sequence fragments of *CaMET1-like1*, *CaCMT3-like*, and *CaDML2-like* without the stop codon were PCR-amplified and subcloned into the Super1300 vector in frame with green fluorescence protein (GFP) driven by the CaMV 35S promoter. Plasmids were transferred into onion epidermal cells using particle bombardment according to Lee *et al.* (2006). GFP fluorescence was detected and captured using a Carl Zeiss LSM 510 system. Each assay was repeated three times. The primers used are listed in Supplementary Table S4.

### Determination of IAA, ABA, and ethylene levels

Levels of auxin (IAA) and ABA were determined in the pericarp of normally developing fruits at the IM, M, T, and R stages and also in premature-ripe and green pericarps of the *CaMET1-like1*-silenced fruit using UPLC-MS (Waters Corporation and ThermoFisher Scientific) according to Wang *et al.* (2017). Ethylene levels of normally developing fruit were measured at the IM, M, T, and R stages using gas chromatography (GC 17A, Shimadzu) according to Xue *et al.*, (2008).

### Virus-induced gene silencing

A VIGS system based on the tobacco rattle virus (TRV) was used to investigate gene function (Liu *et al.*, 2002). Fragments of *CaMET1-like1* (423 bp) and *CaPDS* (452 bp) were PCR-amplified from pepper cDNA and cloned into the pTRV2 vector to generate the plasmids pTRV2*-CaMET1-like1* and pTRV2-*CaPDS*, respectively. pTRV1, pTRV2, pTRV2*-CaPDS*, and pTRV2*-CaMET1-like1* were introduced into *Agrobacterium tumefaciens* strain GV3101 by electroporation. The transformed cells were grown for 8–10 h at 28 °C in Luria–Bertani (LB) medium containing the appropriate antibiotics, and were then collected by centrifugation (5000 *g* for 15 min), re-suspended in infiltration buffer (10 mM MgCl_2_, 10 mM MES, and 100 µM acetosyringone), and incubated at 28 °C for 3 h. pTRV1 and pTRV2 or its derivatives were mixed at a 1:1 (v/v) ratio and infiltrated into cotyledons of 1-week-old pepper seedlings using a 1-cm^3^ syringe without a needle (Liu *et al.* 2002). The infiltrated plants were grown in a culture room at 22 °C, 16/8 h light/dark, 150 μmol m^–2^ s^–1^. Since silencing of *CaPDS* leads to light bleaching of green organs, plants infiltrated with pTRV1+pTRV2-*CaPDS* were used as the positive control to check the activity of pTRV1 and to indicate the spatio-temporal pattern of gene silencing (Guo *et al.*, 2018). When light-bleaching appeared in the positive control, reverse-transcription PCR was conducted to detect pTRV1, pTRV2 and its derivatives in all the infiltrated plants. Plants carrying pTRV1+pTRV2-*CaMET1-like1* and pTRV1+pTRV2 (negative control) were used for further analysis. Flowers at anthesis were hand-pollinated and tagged, and the fruit ripening time was evaluated as the number of days from anthesis to the breaker stage. At least 100 plants were infiltrated with pTRV1+pTRV2*-CaMET1-like1* and at least 30 plants were injected with negative or positive control plasmid mixtures. The whole experiment was repeated twice. The primers used are listed in Supplementary Table S5.

### RNA-seq analysis

Premature-ripe and green pericarps of the *CaMET1-like1*-silenced fruit and red-ripe pericarps of the negative control were collected from the two biological repeats as well as from a preliminary experiment. RNA extraction and library construction were conducted according to Zhong *et al.* (2011). Sequencing was conducted by the Biomarker Technologies Corporation (Beijing, China) on a GAII platform (Illumina) and paired-end reads were generated. Filtration of the reads, alignment, and expression calculations were carried out according to Huang *et al.* (2013). Differentially expressed genes (DEGs) were calculated using DESeq2 in R and were then screened according the following conditions: the average expression value of each gene was greater than 2 fragments per kilobase of transcript per million fragments mapped reads in at least one of the three samples, and the expected false positive rate value was less than 0.05. The KEGG statistics of pathway enrichment were analysed and plotted using BMKCloud (http://www.biocloud.net/) with the default settings. Principal component analysis (PCA) was conducted with expression profiles of all the samples using the stats::prcomp() function in R. Prior to that, data were mean centered and *Z*-scored. The PCA results were plotted and edited using ggplot2::ggplot() (Wickham, 2016) in R and Photoshop CS5, respectively.

### Ripening-related parameters

Soluble solids content (SSC) and titratable acidity (TA) were measured using 50–100 g pericarp tissue taken from 3–5 fruit according to Kong *et al.* (2013). Carotenoids were extracted from 50 mg freeze-dried pericarp samples and measured using aLC-APCI-MS/MS system (AB SCIEX, USA) according to Giuffrida *et al.* (2013). Standard substances examined in study are listed in Supplementary Table S6. Three or more replicates of each assay were performed.

### Phytohormone treatments

Fruit were harvested at the MG stage, surface-sterilized with 2.5% (w/v) NaClO aqueous solution for 15 min, and then washed three times with sterile water. Pericarp discs (radius 0.6 cm) were cut from the equatorial region of the fruit using a hole puncher. The discs were soaked in MES buffer (pH=5.5) containing 5 mmol l^–1^ CaCl_2_, 5 mmol/l^–1^ MgCl_2_, 5 mmol/l^–1^ EDTA, 5 mmol/l^–1^ vitamin C, and 200 mmol/l^–1^ mannitol for 30 mins (Télef *et al.*, 2006). Phytohormones were then added into the buffer to give final concentrations of 25 μM for auxin (IAA; Sigma-Aldrich I5148), 100 μM for ABA (Sigma-Aldrich A1049), 50 μM for gibberellic acid (GA_3_; Sigma-Aldrich G7645), 25 μM for cytokinin (6-BA; Sigma-Aldrich B3408), and 25 μM for ethephon (Sigma-Aldrich 45473). The optimal concentration for each hormone was determined in preliminary experiments. At 4 h after adding the hormones the pericarp discs were collected, frozen with liquid nitrogen, and stored at –80 °C. For each treatment, at least 15 discs were used and the whole experiment was repeated three times. Expression of the DNA methyltransferase and demethylases genes, ripening-related genes (*CaPSY1*, *CaPDS*, *CaPG*, *CaNCED1*, and *CaCYP707A1*) together with several hormone-responsive genes (*CaARF2* for IAA, *CaABI2* for ABA, *CaDELLA* for GA_3_, *CaARR5* for 6-BA, and *CaETR1* for ethylene) were analysed using real-time PCR. Primers used in this experiment are listed in Supplementary Table S3.

### Statistical analyses

Significant differences between two groups of samples were determined using Student’s *t*-test via the t.test function in Excel. Significant differences among multiple groups of samples were tested by ANOVA and *post hoc* Tukey’s HSD test using stats::anova() and agricolae::HSD.test(), respectively, in R (https://CRAN.R-project.org/package=agricolae). For real-time PCR, a significant difference between two groups of samples had to satisfy the following conditions: statistically significant difference at the 0.05 level, and the log_2_-transformed expression fold-change had to be either greater than 1 or less than 0.5.

## Results

### DNA hypomethylation occurs in the UROT of several ripening-related genes during fruit ripening

**Fig. 1.**
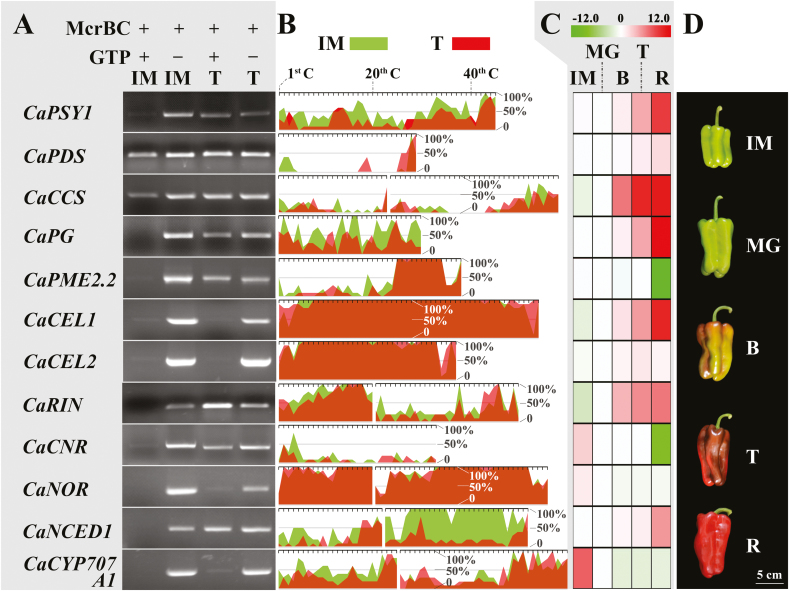
Dynamic changes of DNA methylation and expression levels of ripening-related genes during ripening of pepper fruit. (A) McrBC-PCR analysis of the upstream region of the transcriptional start site (UROT) of ripening-related genes in the pericarp at the immature-green (IM) and turning (T) stages. + and – indicate the presence or absence, respectively, of McrBC and GTP. (B) Bisulfite sequencing of the UROT of ripening-related genes in the pericarp at the IM and T stages. The scale at the top of each chart represents the position of each cytosine in the tested region. (C) Relative expression of the tested genes during pepper fruit ripening. Data were generated by real-time PCR and are log_2_-transformed. For each gene, expression at the MG stage was set as 1. (D) Pepper fruit at five different developmental stages (MG, mature-green; B, breaker; R, red-ripening).

To explore the link between DNA methylation and pepper fruit ripening, the DNA methylation levels of the upstream region of the transcriptional start site (UROT) of several ripening-related genes were determined in the pericarp at the IM and T stages, namely those involved in capsanthin/capsorubin biosynthesis (*CaPSY1*, *CaPDS*, and *CaCCS*), cell wall degradation (*CaPG*, *CaPME2.2*, *CaCEL1*, and *CaCEL2*), ABA metabolism (*CaNCED1* and *CaCYP707A1*) together with genes encoding ripening-related transcription factors (*CaRIN*, *CaCNR*, and *CaNOR*) (Fig. 1A, B). The expression of *CaPSY1*, *CaPDS*, *CaCCS*, *CaPG*, *CaCEL1*, *CaCEL2*, *CaRIN*, and *CaNCED1* was up-regulated during ripening, whereas the expression of the other genes was down-regulated (Fig. 1C, D). According to the results of the McrBC-PCR, DNA hypomethylation was most likely to occur in the UROT of *CaPSY1*, *CaPG*, *CaPME2.2*, *CaRIN*, *CaCNR*, and *CaNCED1* at the T stage (Fig. 1A). In contrast, low levels of DNA methylation were detected in the UROT of *CaPDS* and *CaCCS* at the IM and T stages, whilst consistently high levels of DNA methylation were recorded in the tested regions of *CaCEL1*, *CaCEL2*, *CaNOR*, and *CaCYP707A1* (Fig. 1A). Although the bisulfite-sequencing results were not completely consistent with those of McrBC-PCR, various degrees (~12.42–61.84%) of DNA hypomethylation were recorded in the UROT of *CaPSY1*, *CaCCS*, *CaPG*, *CaPME2.2*, *CaRIN*, *CaCNR*, *CaNCED1*, and *CaCYP707A1* at the T stage (Fig. 1B).

### Identification and expression analysis of DNA methyltransferase and demethylase genes

**Fig. 2.**
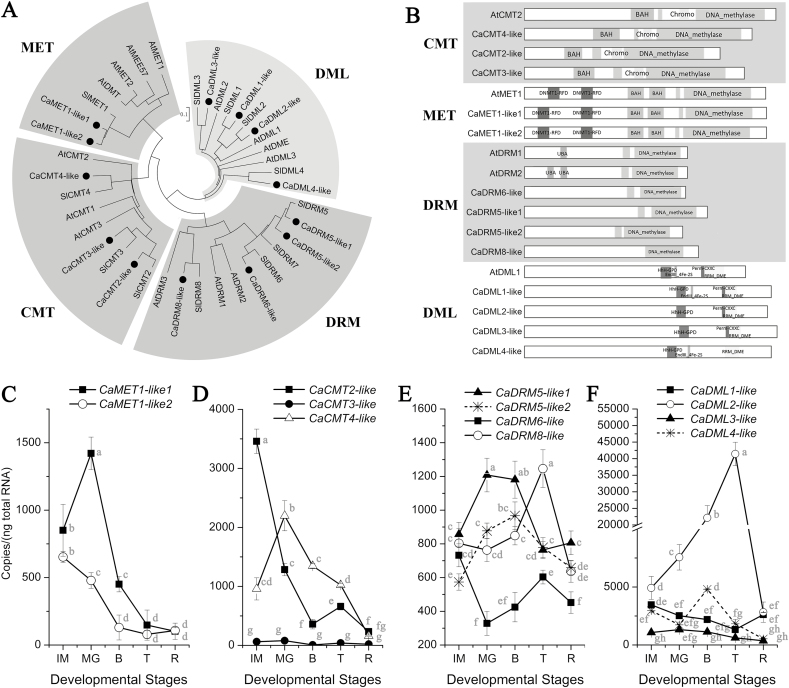
Identification of DNA methyltransferase and demethylase genes in pepper. (A) Phylogenetic tree of DNA methyltransferase and demethylase genes in pepper (Ca), tomato (Sl), and Arabidopsis (At). (B) Conserved domains of the DNA methyltransferase and demethylase genes. (C–F) Dynamic expression changes of *CaMET*s (C), *CaCMT*s (D), *CaDRM*s (E), and *CaDML*s (F) during pepper fruit development and ripening. Significant differences were determined using ANOVA followed by Tukey’s HSD test (*P*<0.05).

Orthologs of Arabidopsis and tomato DNA methyltransferase and demethylase genes were identified in the pepper genome. Based on the phylogenetic tree (Fig. 2A), they were renamed as *CaMET1-like1* (*Capana04g000012*), *CaMET1-like2* (*Capana12g001109*), *CaCMT2-like* (*Capana12g000016*), *CaCMT3-like* (*Capana01g001654*), *CaCMT4-like* (*Capana01g004297*), *CaDRM5-like1* (*Capana02g000460*), *CaDRM5-like2* (*Capana07g000549*), *CaDRM6-like* (*Capana10g002486*), *CaDRM8-like* (*Capana05g002234*), *CaDML1-like* (*Capana09g002341*), *CaDML2-like* (*Capana10g001947*), *CaDML3-like* (*Capana12g002335*), and *CaDML4-like* (*Capana03g000092*). Motif analysis showed that CaCMTs, CaMETs, CaDRMs, and CaDMLs shared conserved functional domains with their Arabidopsis orthologs (Fig. 2B; Supplementary Tables S7, S8), suggesting that they may play similar roles. CaMET1-like1, CaCMT3-like, and CaDML2-like were all localized in the nucleus, which further suggested their potential roles in the regulation of DNA methylation (Supplementary Fig. S2). Expression analysis indicated that both *CaMET1-like1* and *CaMET1-like2* followed a similar pattern of decreased expression after the MG stage; however, at most stages *CaMET1-like1* was expressed at a higher level than *CaMET1-like2* (Fig. 2C). In the *CaCMT* family, expression of *CaCMT2-like* dramatically decreased from the IM to B stage, whereas expression of *CaCMT4-like* peaked at the MG stage and then decreased (Fig. 2D). The expression of *CaCMT3-like* was relatively low during the whole ripening period (Fig. 2D). In the *CaDRM* family, expression of *CaDRM5-like1*, *CaDRM5-like2*, and *CaDRM8-like* was significantly higher than that of *CaDRM6-like* at most stages (Fig. 2E). In particular, *CaDRM5-like1* and *CaDRM5-like2* were highly expressed at the MG and B stages, while expression of *CaDRM8-like* peaked at the T stage (Fig. 2E). In the *CaDML* family, expression of *CaDML2-like*, which peaked at the T stage, was significantly higher than that of the other members at most stages (Fig. 2F). The expression of *CaDML1-like*, *CaDML3-like*, and *CaDML4-like* peaked at the IM, MG, and B stages, respectively. It can be assumed that *CaMET1-like1*, *CaMET1-like2*, *CaCMT2-like*, *CaCMT4-like*, *CaDRM5-like1*, *CaDRM5-like2*, *CaDRM8-like*, and *CaDML2-like* may play important roles in the regulation of DNA methylation during pepper fruit development and ripening.

### Silencing of *CaMET1-like1* promotes fruit ripening and leads to DNA hypomethylation

**Fig. 3.**
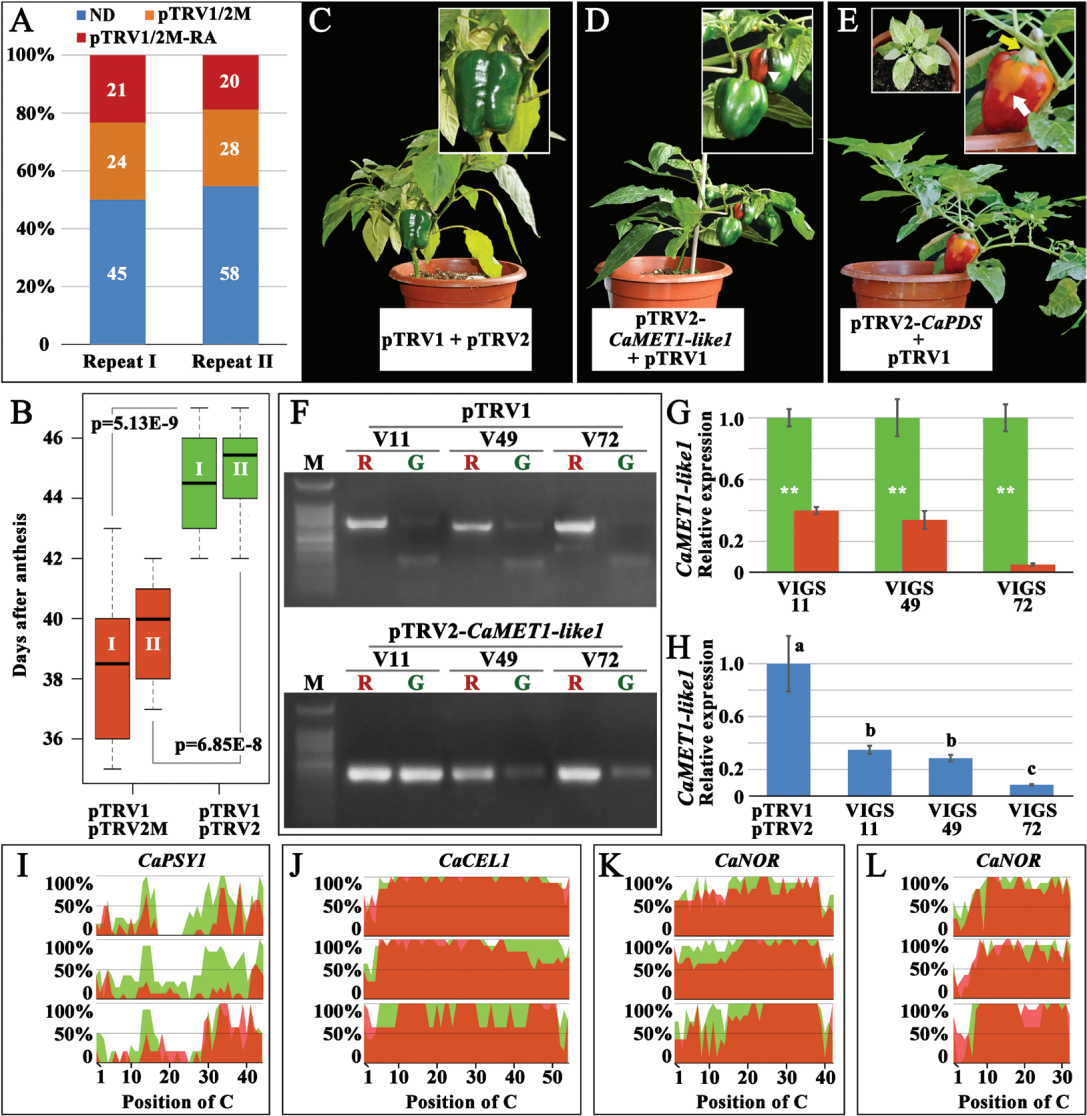
Silencing of *CaMET1-like1* in pepper fruit leads to premature ripening and DNA hypomethylation. (A) Infection efficiency of the virus-induced gene silencing (VIGS). ‘pTRV1/2M-RA’ indicates injected plants that carried both the pTRV1 and pTRV2-*CaMET1-like1* plasmids and which showed accelerated fruit ripening. ‘pTRV1/2M’ indicates injected plants that carried both the pTRV1 and pTRV2-*CaMET1-like1* plasmids and which showed no effect on fruit ripening. ‘ND’ indicates injected plants that only carried one of the above plasmids. (B) Boxplot of the time of onset of ripening of fruit injected with pTRV1 + pTRV2-*CaMET1-like1* (pTRV2M) and pTRV1 + pTRV2. Data are shown from two replicates (I, II). Student’s *t*-test was used to determine the *P*-values. (C–E) Plants injected with pTRV1 + pTRV2 (C), pTRV1 + pTRV2-*CaMET1-like1* (D), and pTRV1 + pTRV2-*CaPDS* (E). The white arrowhead in (D) indicates the coloration on a premature-ripe fruit. The yellow and white arrows in (E) indicate a light-bleached peduncle and pericarp, respectively. Light-bleached leaves are also shown in (E). (F) PCR-based detection of pTRV1 and pTRV2-*CaMET1-like1* plasmids in three heterogeneously colored precocious fruits, which were collected from three replicate injected plants (V11, V49, and V72). ‘R’ and ‘G’ indicate premature-ripe and green pericarps, respectively, collected from the same precocious fruit. (G) Relative expression of *CaMET1-like1* in the premature-ripe (red bars) and green pericarps (green bars). Significant differences were determined using Student’s *t*-test: **P<0.01. (H) Relative expression of *CaMET1-like1* in leaves collected from three replicate plants injected with pTRV1 + pTRV2 and pTRV1 + pTRV2-*CaMET1-like1* (VIGS11, VIGS49, and VIGS72). Significant differences were determined using ANOVA followed by Tukey’s HSD test (*P*<0.01). (I–L) 5mC levels of the upstream region of the transcriptional start site (UROT) of *CaPSY1* (I), *CaCEL1* (J), and *CaNOR* (K, L) in pericarps of premature-ripe (shaded red) and green fruit (shaded green) of V11 (top), V49 (middle), and V72 (bottom) plants. (K) and (L) are two different regions of the UROT of *CaNOR*.

In order to further explore the effect of DNA methylation on ripening, *CaMET1-like1* was silenced using VIGS. In replicates I and II, accelerated fruit ripening was observed in 21 and 20 plants, respectively, which was 4–8 d earlier than that of the negative control (Fig. 3A, B). The color transition in each precocious fruit was restricted to certain areas of the pericarp for several days and led to heterogeneous fruit color (Fig. 3C, D), which was different from that of the untreated fruit (Fig. 1D) and the negative controls (Fig. 3C). Heterogenous coloration was also observed in the *CaPDS*-silenced fruit (Fig. 3E). Later, the premature-ripe and green pericarps were sampled from the precocious fruits produced by the *CaMET1-like1*-silenced plants V11, V49, and V72. RT-PCR indicated that the premature-ripe pericarp simultaneously carried the pTRV1 and pTRV2-*CaMET1-like1* plasmids, whereas the green pericarp only harbored one of them (Fig. 3F). Consistent with this, expression of *CaMET1-like1* in the premature-ripe pericarp decreased to less than 40% of that of the green pericarp (Fig. 3G). Besides the fruit, reduced *CaMET1-like1* expression was also observed in the leaves of the three tested plants (Fig. 3H). Bisulfite sequencing demonstrated that the DNA methylation levels of the UROT of *CaPSY1*, *CaCEL1*, and *CaNOR* were down-regulated in most premature-ripe pericarps (Fig. 3I–L). In accordance with the molecular data, premature-ripe pericarps had a higher level of soluble solids content (SSC) and accumulated more phytoene, β-carotene, zeaxanthin, antheraxanthin, capsanthin, capsorubin, and α-carotene than green pericarps (Tables 1, 2). However, when compared with red-ripe pericarps of the negative control, premature-ripe pericarps accumulated higher levels of phytoene, β-carotene, zeaxanthin, antheraxanthin, violaxanthin, α-carotene, and lutein (Table 2). In contrast, the SSC and carotenoid levels of green pericarps in silenced fruit were similar to those in mature-green fruit of the negative control (Tables 1, 2). With regards to phytohormones, premature-ripe pericarps accumulated more ABA and less IAA than green pericarps (Supplementary Table S9). It can therefore be concluded that silencing of *CaMET1-like1* resulted in premature ripening and DNA hypomethylation in the pepper fruit.

**Table 1.** Soluble solids concentration (SSC) and titratable acids (TA) in pepper fruit samples at different stages of development

Pericarp sample	SSC (%)	TA (ml 1 M NaOH)
Control-MG	1.65±0.15b	6.03±1.38a
Control-R	6.00±0.50a	10.4±2.15a
Silenced-MG	1.85±0.63b	7.13±0.55a
Silenced-R	4.83±1.31a	10.5±2.33a

Control-MG, negative control mature-green fruit; Control-R, negative control red-ripening fruit; Silenced-MG, *CaMET1-like1*-silenced mature-green fruit; Silenced-R, *CaMET1-like1*-silenced premature-ripe.

Different letters indicate significant differences as determined using ANOVA followed by Tukey’s HSD test (*P*<0.01).

**Table 2.** Carotenoid concentrations in pepper fruit samples at different stages of development

Pericarp sample	Phytoene	β-carotene	Zeaxanthin	Antheraxanthin	Violaxanthin	Capsanthin	Capsorubin	α-carotene	Lutein	Capsanthin
Control-MG	0.54±0.15c	26.0±4.59b	5.49±1.14c	7.15±1.32a	56.1±5.20a	0.16±0.02b	ND	1.02±0.24c	107±25.7a	0.16±0.02b
Control-R	4.83±2.76b	18.9±4.57b	15.4±2.09b	ND	7.27±1.54c	26.9±6.20a	5.25±1.09a	0.73±0.05c	0.78±0.03c	26.9±6.20a
Silenced-MG	0.22±0.02c	20.7±1.44b	2.65±0.65c	3.33±0.58b	52.6±14.4ab	0.15±0.01b	ND	1.84±0.16b	93.6±4.20a	0.15±0.01b
Silenced-R	10.8±0.51a	62.2±16.1a	48.5±3.43a	9.63±1.81a	32.2±5.07b	37.0±4.00a	5.61±1.32a	3.82±0.18a	9.25±1.50b	37.0±4.00a

All data are μg g^–1^ fresh weight. ND, not detected.

Control-MG, negative control mature-green fruit; Control-R, negative control red-ripening fruit; Silenced-MG, *CaMET1-like1*-silenced mature-green fruit; Silenced-R, *CaMET1-like1*-silenced premature-ripe.

Different letters indicate significant differences as determined using ANOVA followed by Tukey’s HSD test (*P*<0.01).

### Silencing of *CaMET1-like1* affects gene expression in the fruit

**Fig. 4.**
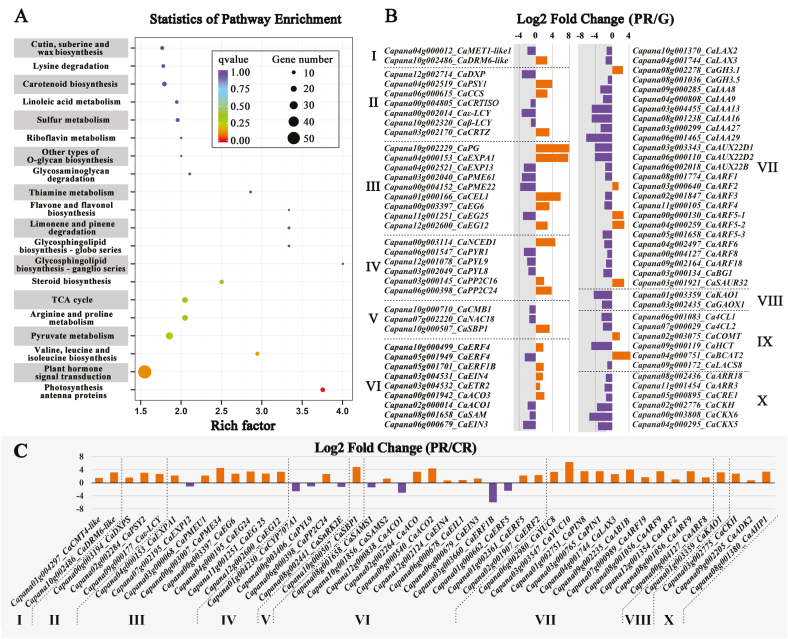
Transcriptome analysis of premature-ripe (PR) and green (G) pericarps of *CaMET1-like1*-silenced pepper fruit and of red-ripe pericarp (CR) of the negative control. (A) Statistics of pathway enrichment of the differentially expressed genes (DEGs) between the premature-ripe and green pericarps. (B) DEGs of interest between the premature-ripe and green pericarps of the *CaMET1-like1*-silenced fruits. (C) DEGs of interest between the premature-ripe and the red-ripe pericarps. The numerals indicate genes involved in (I) ‘DNA methylation’, (II) ‘carotenoids biosynthesis’, (III) ‘cell wall degradation’, (IV) ‘ABA metabolism and signaling’, (V) ‘transcriptional regulation’, (VI) ‘ethylene biosynthesis and signaling’, (VII) ‘auxin biosynthesis and signaling’, (VIII) ‘gibberellin biosynthesis and signaling’, (IX) ‘capsaicin biosynthesis’, and (X) ‘cytokinin biosynthesis and signaling’.

Comparative transcriptional analysis was conducted in order to investigate the effects of *CaMET1-like1* on pepper fruit ripening. PCA showed that samples collected from red-ripe pericarps of the negative control and from premature-ripe and green pericarps of the silenced fruit were clearly separated in the plot defined by PC1 and PC2, which together explained ~80% of the variation (Supplementary Fig. S3), suggesting that gene silencing affected the pepper fruit transcriptome. DEGs between the premature-ripe and green pericarp were mainly enriched in the categories of plant hormone signal transduction, pyruvate metabolism, arginine and proline metabolism, the TCA cycle, and steroid and cutin biosynthesis (Fig. 4A).DEGs between the premature-ripe pericarp and the red-ripe negative control pericarp were mainly enriched in phenylpropanoid biosynthesis, photosynthesis, DNA replication, porphyrin, and chlorophyll metabolism (Supplementary Fig. S4). Putative ripening-related DEGs were extracted from the two comparisons, and included those involved in DNA methylation, carotenoid biosynthesis, cell wall degradation, phytohormone (ABA, ethylene, auxin, GA, and cytokinin) metabolism and signaling, auxin polar transport, transcriptional regulation, and capsaicin biosynthesis (Fig. 4B, C). In particular, compared with the genes in the green pericarp of the silenced fruit, in premature-ripe pericarps *CaMET1-like1* was down-regulated while *CaDRM6-like* was up-regulated; *CaPSY1* and *CaCCS*, which are involved in capsanthin/capsorubin biosynthesis, were up-regulated while *Caε-LCY* and *Caβ-LCY* were down-regulated; and *CaPG*, *CaEXPA1*, and *CaCEL1*, which are involved in cell wall degradation, were up-regulated. With regards to ethylene biosynthesis genes, *CaACS10* and *CaACO1* were down-regulated while *CaACO3* was up-regulated. For auxin-related genes, most of the *CaLAX*s, *CaIAA*s, *CaAUX*, and *CaARF*s were down-regulated, except for *CaGH3.1*, *CaARF2*, *CaARF5-1*, and *CaARF5-2*, which were up-regulated; *CaKAO1* and *CaGAOX,1*, which are involved in GA metabolism, were down-regulated. For capsaicin biosynthesis genes, expression of *CaCOMT* and *CaBCAT2* was increased, while expression of *Ca4CL1/2*, *CaHCT*, and *CaLACS8* was decreased. *CaARR3/18*, *CaCRE1*, *CaCKH*, and *CaCKX5/6*, which are involved in cytokinin metabolism, were down-regulated. Transcription factors *CaCMB1* and *CaNAC18* were down-regulated, whereas *CaSBP1* was up-regulated (Fig. 4B). In another comparison, *CaDRM6-like* and *CaCMT4-like* were up-regulated in premature-ripe pericarps relative to red-ripe pericarps, whilst *CaMET1-like1* was not identified as a DEG. *CaDXPS*, *CaPSY2*, and *Caε-LCY*, which are involved in carotenoid metabolism, were up-regulated. With regard to cell wall-related genes, expression of *CaEXPA1*, *CaPMEU1/34*, and *CaEG6/12/24/25* was increased, and expression of *CaEXP12* was decreased. *CaCYP707A1* (involved in ABA degradation), *CaPYL9*, and *CaSnRK2E* (both participants in ABA signaling) were all down-regulated, while *CaPP2C24* was up-regulated. Regarding ethylene biosynthesis genes, expression of *CaSAMS2* and *CaACO/2* was increased, whilst expression of *CaSAMS1* and *CaACO1* was decreased; *CaEIN3/4*, *CaEIL1*, and *CAERFID2/5*, which are involved in ethylene signaling, were up-regulated, while expression of *CaERF1B/5* was down-regulated. Genes participating in auxin metabolism (*CaYUC8/10*), signaling (*CaARF2/8/9/19*), and polar transport (*CaPIN1/8* and *CaLAX3*), GA (*CaKAO1*) and cytokinin metabolism (*CaCKH*, *CaADK2*, and *CaAHP1*), and transcriptional regulation (*CaSPB1*) were all up-regulated (Fig. 4C).

### Phytohormones affect the expression of genes related to DNA methylation and ripening in the pericarp

**Fig. 5.**
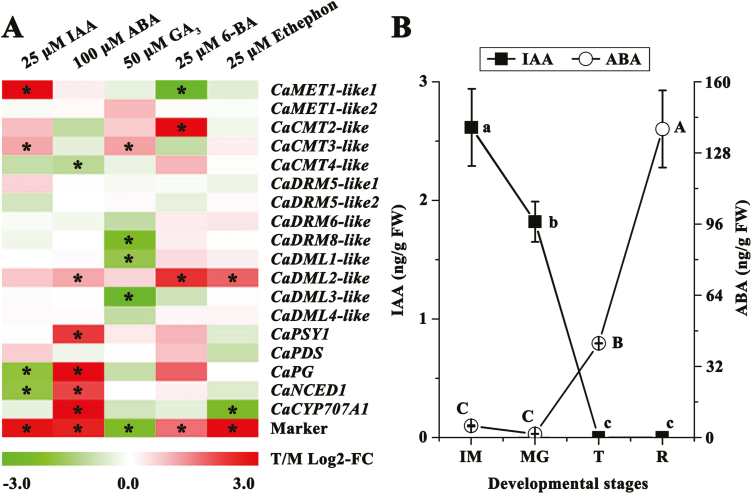
Responses of genes to phytohormones in mature-green (MG) pericarps and dynamic changes in endogenous ABA and IAA during pepper fruit ripening. (A) Changes in gene expression in response to exogenous auxin (IAA), ABA, gibberellin (GA_3_), cytokinin (6-BA), and ethephon in pericarp discs at the MG stage. Data are log_2_-transformed. ‘T/M’ indicates the treatment value divided by the mock control value. ‘*’ indicates that the log_2_ fold-change (FC) value is either greater than 1 or less than –1. (B) Dynamic changes of ABA and IAA levels during pepper fruit ripening. ‘IM’, immature-green; ‘M’, mature-green; ‘T’, turning; ‘R’, red-ripening. Different letters indicate significant differences as determined using ANOVA followed by Tukey’s HSD test (*P*<0.01): IAA, lowercase letters; ABA, uppercase letters.

In order to investigate the relationships between phytohormones and genes related to DNA methylation during pepper fruit ripening, phytohormones were applied exogenously to discs of mature-green pericarp. Exogenous IAA clearly up-regulated the expression of *CaMET1-like1* and *CaCMT3-like*, but down-regulated the expression of *CaPG* and *CaNCED1* (Fig. 5A). Exogenous ABA repressed the expression of *CaCMT4-like*, but induced the accumulation of transcripts of *CaDML2-like*, *CaPSY1*, *CaPG*, *CaNCED1*, and *CaCYP707A1*. Gibberellin down-regulated the expression of *CaDRM8-like*, *CaDML1-like*, and *CaDML3-like*, but up-regulated the expression of *CaCMT3-like*. Application of 6-BA, a synthetic cytokinin, resulted in up-regulation of *CaCMT2-like*, *CaDML2-like*, and *CaPG*, and in down-regulation of *CaMET1-like1*. Ethephon clearly induced the expression of *CaDML2-like*, but repressed the expression of *CaCYP707A1*.

### Dynamic changes of IAA, ABA, and ethylene levels during ripening

In order to examine the effects of IAA and ABA on pepper fruit ripening, their concentrations were measured at different developmental stages. A decrease in endogenous IAA was observed from the IM to T stage (Fig. 5B). In contrast, the levels of endogenous ABA decreased slightly from the IM to MG stage and then increased and peaked at the R stage. No ethylene signal was detected at any of the tested stages (Supplementary Fig. S5).

## Discussion

### DNA methylation may be involved in the regulation of ripening in pepper fruit

DNA methylation has been recently been discovered to regulate fruit ripening in tomato, strawberry, and sweet orange (Liu *et al.*, 2015; Lang *et al.*, 2017; Cheng *et al.*, 2018; Huang *et al.*, 2019). In our present study, DNA hypomethylation was observed in the UROT of some ripening-related genes during pepper fruit ripening (Fig. 1A, B). Consistent with this, dynamic changes in the expression of DNA methyltransferase and demethylase genes associated with ripening were also observed (Fig. 2C–F), suggesting that there was very likely a link between DNA methylation and fruit ripening. Moreover, silencing of *CaMET1-like1* using VIGS resulted in DNA hypomethylation (Fig. 3I–L), premature accumulation of carotenoids (Table 2), increased levels of SSCs and ABA (Table 1, Supplementary Table S9), and decreased levels of IAA (Supplementary Table S9). The gene expression profile of premature-ripe pericarps of the *CaMET1-like1-*silenced fruit was also shifted towards to that of the red-ripe pericarps of the negative control (Supplementary Fig. S3). In particular, capsanthin/capsorubin biosynthesis (up-regulation of *CaPSY1* and *CaCCS*), cell wall degradation (up-regulation of *CaPG*, *CaEXPA1*, and *CaCEL1*; down-regulation of *CaEXP13*, *CaPME22/61*, and *CaEG25*), and ABA biosynthesis (up-regulation of *CaNCED1*) were promoted, whereas IAA signaling (down-regulation of *CaIAA8/9/13/16/27/29* and *CaAUX22B/D1/D2*) and gibberellin biosynthesis (down-regulation of *CaKAO* and *CaGAOX1*) were repressed (Fig. 4B). Therefore, it can be deduced that *CaMET1-like1*, which is involved in the regulation of DNA methylation and hypomethylation in pepper fruit, may promote ripening. In accordance with this, ripening-associated DNA hypomethylation is also observed in tomato and strawberry, and its disturbance leads to abnormal fruit ripening (Liu *et al.*, 2015; Lang *et al.*, 2017; Cheng *et al.*, 2018). This suggests that the regulation of DNA methylation in fleshy fruit ripening may be common in some species. DNA hypomethylation occurs in the putative promoter region of *PSY1* in tomato and pepper and it enhances the expression of the gene (Liu *et al.*, 2015; Lang *et al.*, 2017; Fig. 1A, B), suggesting that *PSY1* may be a key point in the regulation of fruit ripening by DNA methylation.

The comparison between premature-ripe and red-ripe pericarps was also informative. Compared with red-ripe pericarps, the premature-ripe ones accumulated more phytoene, β-carotene, zeaxanthin, antheraxanthin, violaxanthin, α-carotene, and lutein (Table 2), whereas the levels of capsanthin and capsorubin levels were not changed significantly. A possible reason for this was the higher expression of *CaDXPS*, *CaPSY2*, and *Caε-LCY* in premature-ripe pericarps. The up-regulation of the first two genes may have led to the increase of total carotenoids, while the increased expression of *Caε-LCY* may have enhanced the δ-carotene–α-carotene–lutein pathway and ultimately led to high levels of α-carotene and lutein. However, since the expression of *CaCCS* and *CaVDE* was not included in the list of DEGs (Fig. 4B), the capsanthin and capsorubin levels may not have been significantly changed.

### Transcriptional regulation of DNA methylation during ripening

The DNA methylation level is controlled by DNA methyltransferases (MET1, CMT2, CMT3, and DRMs) and demethylases (ROS1, DME, and DMLs) (Deleris *et al.*, 2016; Gallusci *et al.*, 2016). In our study, members of these families were identified in the pepper genome (Fig. 2A), which is consistent with the hypothesis that DNA methylation is conserved in all flowering plants (Goll and Bestor, 2005). At the transcriptional level, expression of *CaMET*s and *CaCMT*s showed dynamic changes associated with ripening (fold-change >2), whereas expression of *CaDRM*s was barely altered (fold-change <2), especially after the MG stage (Fig. 2C–F), suggesting the *CaMET*s and *CaCMT*s may mainly control the maintenance and *de novo* methylation of cytosine, respectively, during pepper fruit ripening. Moreover, most of the *CaMET*s and *CaCMT*s were down-regulated after the MG stage (Fig. 2C, D), suggesting decreased DNA methylation activity. However, similar to tomato, pepper fruit generally undergo genome endoreduplication after the MG stage (Cheniclet *et al.*, 2005; Chevalier *et al.*, 2007; Ogawa et al., 2010, 2012). Therefore, passive DNA demethylation is highly likely to occur during pepper fruit ripening. On the other hand, expression of *CaDML2*, an ortholog of *ROS1* (*AtDML1*) and the highest expressed member of the *CaDML* family, peaked at the T stage (Fig. 2F), suggesting that an active demethylation process may be occurring during pepper fruit ripening. Thus, it seems that the ripening-associated DNA hypomethylation in pepper may be mainly controlled by *CaMET*s, *CaCMT*s, and *CaDML2* at the transcriptional level. Similar phenomena have been observed in tomato, in which the DNA hypomethylation associated with ripening is controlled by the expression of both DNA methyltransferase and demethylase genes (Teyssier *et al.*, 2008; Liu *et al.*, 2015; Lang *et al.*, 2017; Yang *et al.*, 2019). However, in strawberry and sweet orange, changes in ripening-associated DNA methylation are only attributed to variations in the expression of RdDM-related and DNA demethylase genes, respectively (Cheng *et al.*, 2018; Huang *et al.*, 2019). Therefore, it can be concluded that the transcriptional regulatory mechanism of the variations in DNA methylation that are associated with ripening may be not the same among different species.

### Interactions between phytohormones and DNA methylation during ripening

**Fig. 6.**
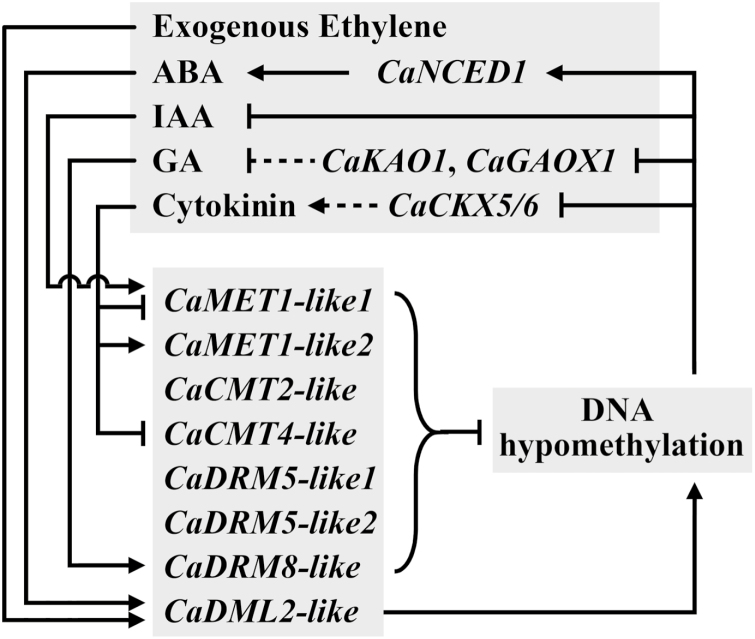
A proposed model of the interactions between DNA methylation and phytohormones during ripening of pepper fruit. Arrows indicate promotion, blocked lines indicate inhibition. Solid lines indicate that the interaction is supported by data obtained from this study. Dashed lines indicate that the interaction is deduced from the gene function.

Ethylene is considered to be a positive regulator of climacteric fruit ripening (Oeller *et al.*, 1991; Lanahan *et al.*, 1994; Li *et al.*, 2007; Lee *et al.*, 2012; Sekeli *et al.*, 2014). However, in this study, no endogenous ethylene burst was detected during fruit ripening (Supplementary Fig. S5), suggesting that our pepper inbred line harbors non-climacteric fruit. Consistent with this, compared with green pericarps of the silenced fruit, *CaACS* was not up-regulated in premature-ripe pericarps (Fig. 4B). A similar phenomenon was also observed by Aizat *et al.* (2013). This suggests that an endogenous ethylene burst is not indispensable for the non-climacteric ripening of pepper fruit. In accordance with this, expression of *CaPSY1*, *CaPDS*, and *CaPG* did not clearly respond to short-term treatment with ethephon (Fig. 5A), suggesting that these genes may not be directly regulated by exogenous ethylene. However, a previous study determined that continuous treatment with exogenous ethylene promoted non-climacteric fruit ripening (Fox *et al.*, 2005), suggesting that exogenous ethylene may have some slow or indirect effects on the ripening of this type of fruit. In our study, expression of *CaDML2-like* was clearly induced by short-term ethephon treatment (Figs 5A, Fig. 6), suggesting that this gene and even DNA methylation may be involved in the process.

ABA has been widely accepted as an important regulator of non-climacteric fruit ripening (Deruère *et al.*, 1994; Jia *et al.*, 2011; Leng *et al.*, 2014; Wang et al., 2013, 2017). Consistent with this, we found that exogenous ABA promoted carotenoid biosynthesis, cell wall degradation, and DNA hypomethylation through altering the expression of *CaPYS1*, *CaPG*, and genes related to DNA methylation (*CaDML2-like* and *CaCMT4-like*), respectively (Figs 5A, Fig. 6). Endogenous ABA dramatically increased from the MG to R stage (Fig. 5B), suggesting a positive role in the regulation of pepper fruit ripening. ABA levels as well as the expression of *CaNCED1* and two *CaPP2C*s were up-regulated in premature-ripe pericarps (Supplementary Table S9, Figs 4B, Fig. 6). *CaNCED1* encodes a key enzyme in ABA biosynthesis (Schwartz *et al.*, 1997; Tan *et al.*, 1997; Burbidge *et al.*, 1999). *CaPP2C* encodes a negative component of ABA signaling and is usually up-regulated by ABA (Ma *et al.*, 2009; Sun *et al.*, 2010; Wang *et al.*, 2015). Therefore, it can be deduced that ABA biosynthesis and signaling were activated by DNA hypomethylation in the non-climacteric pepper fruit. Similar phenomena have also been observed in strawberry, in which ABA biosynthesis genes are up-regulated during fruit ripening whilst at the same time undergoing a loss of DNA methylation in their 5´- and 3´-regulatory regions (Liao *et al.*, 2018; Cheng *et al.*, 2018). Hence, it can be concluded that ABA and DNA methylation may interact with each other at the transcriptional level during pepper fruit ripening.

Auxin is often considered as a repressor of non-climacteric fruit ripening that acts fully or partially through antagonizing the effects of ABA (Manning *et al.*, 1994; Davies *et al.*, 1997; Liu *et al.*, 2005; Böttcher *et al.*, 2011; Symons *et al.*, 2012; Ziliotto *et al.*, 2012; Liao *et al.*, 2018). In accordance with this, we found that exogenous IAA inhibited cell wall degradation, ABA biosynthesis, and DNA hypomethylation through affecting the expression of *CaPG*, *CaNCED1*, and DNA methyltransferase genes (*CaMET1-like1* and *CaCMT3-like*), respectively (Figs 5A, Fig. 6). Moreover, endogenous IAA levels decreased from the IM to T stage, suggesting that it may be involved in the regulation of ripening of the non-climacteric pepper fruit. In premature-ripe pericarps, the endogenous IAA level as well as most of the differentially expressed *AUX/IAA* genes were down-regulated (Supplementary Table S9, Fig. 4B). *AUX/IAA* genes are important elements of auxin signaling and positively respond to auxin treatment (Waseem *et al.*, 2018). Therefore, it can be concluded that DNA hypomethylation caused by the silencing of *CaMET1-like1* led to the inhibition of auxin signaling and a decreased level of auxin.

Generally, gibberellin (GA) and cytokinin are considered to be suppressors of fruit ripening (Dostal and Leopold, 1967; Biale, 1978; Deruère *et al.*, 1994; Martínez *et al.*, 1994; Martínez-Romero *et al.*, 2000; Alós *et al.*, 2006; Kader, 2008; Peppi and Fidelibus, 2008). However, in our study, exogenous GA_3_ and 6-BA did not significantly affect the expression of *CaPDS*, *CaPSY1*, *CaPG*, *CaNCED*, or *CaCYP707A1* (Fig. 5A), indicating that under our experimental condition these two hormones had weak effects on pepper fruit ripening. In tobacco leaves, GA is considered as a determinant for global DNA hypomethylation (Manoharlal et al., 2018*a*, 2018*b*). However, in our study, exogenous GA_3_ and 6-BA did not show clear effects on DNA methylation at the transcriptional level (Figs 5A, Fig. 6). DNA hypomethylation seemed to inhibit GA accumulation at the transcriptional level, since the expression levels of *CaKAO1* and *CaGAOX1* were lower in premature-ripe pericarps than in green pericarps of *CaMET1-like1*-silenced fruit (Fig. 4B, Fig. 6). With regard to cytokinin, DNA hypomethylation was likely to inhibit its degradation by down-regulating the expression of *CaCKX5/6* (Figs 4B, Fig. 6).

### Conclusions

A hypothetical model is proposed (Fig. 6) that indicates a high likelihood of a role for DNA methylation in the regulation of fruit ripening in non-climacteric pepper, which is mainly controlled by *CaMET1-like1*, *CaMET1-like2*, *CaCMT2-like*, *CaCMT4-like*, and *CaDML2-like* at the transcriptional level. Silencing of *CaMET1-like1* by VIGS results in DNA hypomethylation and premature fruit ripening, which includes the accumulation of carotenoids, increased levels of SSC and ABA. and decreased levels of IAA. Under *in vitro* conditions, ABA promotes DNA hypomethylation in the pericarp at the MG stage at the transcriptional level, whereas IAA inhibits this process. Exogenous gibberellin and cytokinin show indistinct roles in DNA methylation in MG pericarps of non-climacteric pepper fruit.

## Supplementary data

Supplementary data are available at *JXB* online.

Table S1. Primers used for McrBC-PCR.

Table S2. Primers used for bisulfite sequencing.

Table S3. Primers used for real-time PCR.

Table S4. Primers used for subcellular localization plasmid construction.

Table S5. Primers used for VIGS construction and detection.

Table S6. Standard substances used in study.

Table S7. IDs and conserved domains of pepper DNA methyltransferase genes.

Table S8. IDs and conserved domains of pepper DNA demethylases genes.

Table S9. ABA and IAA levels in premature-ripe and green pericarps of *CaMET1-like1*-silenced fruit.

Fig. S1. Upstream regions of transcriptional start sites for McrBC-PCR and bisulfite sequencing.

Fig. S2. Subcellular localization of *CaDML2-like*, *CaMET1-like1*, and *CaCMT3-like* in onion epidermal cells.

Fig. S3. PCA of the RNA-seq data.

Fig. S4. Enrichment of DEGs between premature-ripe pericarps of *CaMET1-like1*-silenced fruit and red-ripe pericarps of negative control fruits.

Fig. S5. Ethylene production of fruit at different developmental stages.

eraa003_suppl_supplementary_tables_S1_S9_figures_S1_S4
