# Supplementary material for: DNA methylation is involved in the regulation of pepper fruit ripening and interacts with phytohormones
Source: J Exp Bot. 2020 Jan 7;71(6):1928–42. doi: 10.1093/jxb/eraa003 (PMC7242076; doi:10.1093/jxb/eraa003)
Supplement: eraa003_suppl_supplementary_tables_S1_S9_figures_S1_S4 [file eraa003_suppl_supplementary_tables_s1_s9_figures_s1_s4.pdf]

Supplementary Files

Table S1-S9

Table S1. Primers for McrBC-PCR

Gene ID	Gene symbol	Sequence
Capana02g001917	*CaCNR*	F: ACATCACAAATTCACAGTTTCTCG
		R:GCAAGTATCGCTATTTGGTAGTCTT
Capana07g002220	*CaNOR*	F: CCCTAACAAGTTGTGGCTATTCTTG
		R:ACCAATTAGAGTATCTACCCGCTCC
Capana11g002005	*CaRIN*	F: GACTCCACACTAAATGCTTGAAACG
		R:TCATTCATACCAACCACACCATTCT
Capana04g001637	*CaCYP707A1*	F: TAACTAACATGAAGTGCCAAAGGGA
		R:AATTTAGCCAATTTGCCATTGGA
Capana00g003114	*CaNCED1*	F: GTTTTGACAGCATAAATGCAGAATC
		R:GCGTTCGTTATAGAAGAGTGTCTCC
Capana06g000615	*CaCCS*	F: CCTAAGTGTGTATCTATATCCTCCG
		R:CAGTGAGGCTTGAAGTAGCAGAG
Capana04g002519	*CaPSY1*	F: TGAGCTGATTTATTCTGAATCACTC
		R:CACACACTACCAGAACAATAGAAAG
Capana03g000054	*CaPDS*	F: TGTGTTCAAATAAGACTTTCCTTTG
		R:GTTGTTTAATACTACATCGCCTCAA
Capana01g000166	*CaCel1*	F: AAAGAGCACGGGTTCATATGCC
		R:ACGTGAGAATGATTCGTGTGCG
Capana01g003323	*CaCel2*	F: TATCCTATCAAAATAGGTGTCATGT
		R:CTGAATGCATCTCAATTGTATCCA
Capana10g002229	*CaPG*	F: YTATAGTTGAAAAGTYAAATYYAYA
		R:ARTCRAARCCACACCTAAACTATAC
Capana00g004152	*CaPME2.2*	F: CATACTGGCCACATGATGGTATG
		R:TTCACTCGTACTTCTCTCACAGAAT

Table S2. Primers for bisulfite sequencing

Gene ID	Gene symbol	Sequence
Capana02g001917	*CaCNR*	F1: GYAYGAGGAATATTYAAATGAGYTA
		R1:ACARCTCTRTRRCTATTARCCATAT
		F2:ATTYATATGGYTAATAGYYAYAGAG
		R2:CAATRAACTARCCCTTTATRTRTTT
Capana07g002220	*CaNOR*	F1:TTTYGTYAAATAAGGTTYAGATGAA
		R1:TRCTCCATCTTTAACATRARRTATC
		F2:ATYGTATAAGTTGAGTYTYGAYAYA
		R2:CAAAAAATCTCAACTCTCTRAACAT
Capana11g002005	*CaRIN*	F1:YTAAATGYTTGAAAYGTGGAATTTG
		R1:RAATRTACRTARATCTTACCATTACCT
		F2:AATGGTTGTYYYAYAATAGTTGAYT
		R2:TCATTCATACCAACCACACCATTCT
Capana00g003114	*CaNCED1*	F1:TATYTTTGAYYAYGAAGTTGGTTY
		R1:AATCCATAACTATATRCATRCTARA
		F2:TAGYATGYATATAGTTATGGATTAG
		R2:TAAARRACTTRTCAARCTATCATCA
Capana04g001637	*CaCYP707A1*	F1:TTYGTYGAATTTGGGTTTATYGY
		R1:ATTRTCRARTTCAAATCCAARTARC
		F2:YTTYTTTTYATAGGGAAGTAATTGY
		R2:CTACTRACCTCTATTRARTCTCATR
Capana01g003323	*CaCCS*	F1:AGGAAYTAGAGTTYGGATTYAATAG
		R1:ACCAATTTCAAACTCATACTARACC
		F2:ATATYYTYYGAGAATTTGGAATTTG
		R2:ARACRARCATTTCCAAARTCTACCA
Capana06g000615	*CaPSY1*	F1:AGTTGYAGTYTTTGAYAATYTGTGT
		R1:CACACACTACCARAACAATARAAARTT
		F2:AGGTTTGTTTYAYTTYAATTYTTGT
		R2:TRATRTTCATRAATTCTRAACTCTA
Capana03g000054	*CaPDS*	F: AYAAGAYGGTTAAATGTTAGGTAYA
		R:TATAARCTRRAAATATRTAATCTTC
Capana10g002229	*CaPG1*	F: TAGYTTGTATGATAGAGGTTAAATG
		R:ACARATRRACATTACAAATTTAATT
Capana01g000166	*CaCel1*	F: TTTGGAAYAAYYGTTAGTATGAAGA
		R:TRCTCTTCAACTARTCAACTARCTC
Capana01g003323	*CaCel2*	F: AGYACAYYYAAAGTAGTGAAAAAAT
		R:CACATRCAAARARAAATRTRGACTTA
Capana00g004152	*CaPME2.2*	F: TTYATAAGTAYGTTAYATYGYTTAA
		R:AARTCTACTTCCAARTCTCATCATR

Note: “Y” and “R” indicates “C/T” and “G/A” , respectively.

Table S3. Primers for real-time PCR

Gene ID	Gene symbol	Sequence
Capana01g004297	*CaCMT2-like*	F: ACCAAACTCTCTGGTGTTAAGCTTGC
		R:TTCACCTTCTTCTCGGTCGTCTACC
Capana12g000016	*CaCMT3-like*	F: CGAAGCTGGTGAAGGCATGAGT
		R:GCAGACGCCAAGCATAATGTGAC
Capana01g001654	*CaCMT4-like*	F: GCCTCATCGCTACATTAGAAAGGACA
		R:GTAAAATACCGCACACCATCGACAG
Capana09g002341	*CaDML1-like*	F: CGTCCAGGCAGAACAAGCTAACATC
		R:TCGTCGCTAACTGAAATGATCATCC
Capana10g001947	*CaDML2-like*	F: TGCACTGAACATTCACAAGAAGGTGA
		R:AAGGCTGTGGCTTCTGTAACTTGCT
Capana12g002335	*CaDML3-like*	F: CAGTTGCCGCAGTCATACCTATGCT
		R:CAATGTCGCTTGCTAATAGGTCTGG
Capana03g000092	*CaDML4-like*	F: TGATGATCAGCATTTAGAGATGGCA
		R:TACACCATCAGAGTGCATCCGAAAC
Capana10g002486	*CaDRM6-like*	F: GCCAAGGCTAAGGTTATTCTTTGCA
		R:CAGTTTCTCCCACTCCCAATCAACTC
Capana02g000460	*CaDRM5-like1*	F: TGTTCGGATATTGGACTTGGTCAAG
		R:GGAGAAACGGTCCTCTGTATGGTTG
Capana07g000549	*CaDRM5-like2*	F: AACAACCTTGCAGGCAGTAACAGGG
		R:TGATGGCTTTATCCCTTGATGTCTTG
Capana05g002234	*CaDRM8-like*	F: GGTATCCTTCAGGTGACAGACAGCG
		R:TGGAAGCAATGTTTCAGCGATAGAAG
Capana04g000012	*CaMET1-like1*	F: CAGAAGCACAGGCAAATAGGAAATG
		R:CACTCCTAAATATGGGCGTACATGAAG
Capana12g001109	*CaMET1-like2*	F: CACAAGCACAGGCAAATAGGAAATG
		R:GTAACATTTGCTCCTTTGTCAATGTGA
Capana06g000615	*CaCcs*	F: GATGTTGATCCCAAGTACTGGCACG
		R:GGCTCTCTATTGCTAGATTGCCCAG
Capana01g000166	*CaCel1*	F: CACCATAGAGCCTCATCACTTCCTT
		R:GACCAATCAAAGCGGCTACGGAT
Capana01g003323	*CaCel2*	F: AATCGATTGATTGGTGCTGTTGTTG
		R:GAGCAGACCATGGATGTTGTGTTGT
Capana04g001637	*CaCYP707A1*	F: GATCCTTCAAGATTTGAGAATGCGC
		R:CTGCTAGTCCACCTACTGGGACAGG
Capana00g003114	*CaNCED1*	F: GTGGCTAACTTTGCAAACTATGGCT
		R:CCAGAAATACCAGCTGAGCTCCAAT
Capana03g000054	*CaPDS*	F: ATGTTGGAATTGGTCTTTGCGC
		R:ACAGGGTTCACAACCTGGCACAGT
Capana10g002229	*CaPG*	F: CAAAGGCACAAGTGCAACAGATGT
		R:TGCAGGACAATATGGTGAAACATGT

Capana04g002519	*CaPSY1*	F: TGGAAGAGTGACCGATAAATGGAGA
		R:GCAATCAACTTCTTTGGTTTGCTCA
Capana02g001917	*CaCNR*	F: AGCGATGTGGTTTAAGCATGCTCTC
		R:ACTTGTGAGCACCATGTGATATGCA
Capana07g002220	*CaNOR*	F: TTCTCGGCTCTCTTAGTGACGGAGT
		R:CCAAATCCAGCAATCCCACTAATCT
Capana11g002005	*CaRIN*	F: CAAAGTTGGAAGAACTTGGTGTTGC
		R:CAAAGCATCCATCCAGGTACAACTC
Capana00g004152	*CaPME2.2*	F: TGGTTGAGTTCTACCGGCGTGAC
		R:TGAACACGGGATGCTTAGTACAGCC
Capana06g002873	*CaUBI3*	F: CACATGTACAAGACTGACAGGGCCA
		R:AGACCCGTTCCTTGACAACCCAC
Capana00g000688	*CaACCO3-like1*	F: TGCTCAAACAGACGGGACTAGGATG
		R:GCACTTGCAATTGGATCAACTTTCA
Capana00g001942	*CaACCO3-like2*	F: ATCCAGGAAACGATGCAGTGATCTA
		R:GCACTTGCAATTGGATCAACTTTCA
Capana01g000451	*CaACS10-like1*	F: GCTTCTACTGCTGGGCTGACATGAG
		R:CATAACCTGAACCATCCAGGTTCAA
Capana02g000014	*CaACCO-like1*	F: AAGCAATGAAGACTCTGGAAACTGC
		R:GAAAACCCAAGTTGCACAAAGCAA
Capana02g002264	*CaACCO-like2*	F: TGATTGCTCAACAAGATGGCACTAG
		R:AACCTAGGCTCCTTAGCCTGGAATT
Capana03g002485	*CaACS12-like1*	F: AGGAGAGCTTGAGCTGTGGGAGAAG
		R:TCAGGAGGCCTAACTACGTTCAACA
Capana08g000980	*CaACS2-like1*	F: TCGCGAATATGGATGATGGAACAGT
		R:GGAATTGGTGACGAAAGTGGTGACA
Capana09g000540	*CaACS2-like2*	F: AGATTTGAAGCCATGAAAGCTATGG
		R:AAGAAGGAACACAACTCATTTTGGG
Capana01g000175	*CaACS-like*	F: CTCGCTAGGATTCGGATGTTCGTG
		R:CTCGTACAAGCGGCGAGATAGGAA
Capana03g000640	*CaARF2*	F: TGTCAGAGTAAGAGTTGGCTGGTTG
		TCTTCCCTTCTTTAGCATCTGAGCC
Capana03g000145	*CaABI2*	F: GAATGGACATCGTGTATTTGGCGT
		R:GAATTCTTCGCCTAGCCACTTCACA
Capana11g001454	*CaARR5*	F: TCGGACACATGATCGATGATATCGT
		R:CCATATCCCGTTATTCAATTCCCAT
Capana00g004595	*CaETR1*	F: TCATCATGAACGTAGGAGTTGGACG
		R:CATCCTTGCATCGCATTTAATCAGA
Capana03g000088	*CaDELLA*	F: ATGTCGGAGGTGTATCTAGGGCGTC
		R:TTGTATGCCAACCCAACATCAAACA

Table S4. Primers for subcellular localization plasmid construction

Gene ID	Gene symbol	Sequence
Capana10g001947	*CaDML2-like*	F: ATGGACTCATGGATCCCAGCA
		R:GGAGGCTACTCCTTTGTCTTCATTT
Capana04g000012	*CaMET1-like1*	F: ATGGGTTCTCTGGCGGCG
		R:AGTGGGCCTCCTCTTACTCTCAACA
Capana12g000016	*CaCMT3-like*	F: ATGCCGAGTAAACGTAAAGATTCTC
		R:ATTATTGGACACTTCGTTACAACTC

Table S5. Primers for VIGS construction and detection

Gene ID	Gene symbol	Sequence
Capana04g000012	*CaMET1-like1*	F: GTTGTCAAACGAGGTGGTTGATTG
		R: CCACCTCTCTCTCATTAGCTGCATTCC
	TRV2	F: TGTTACTCAAGGAAGCACGAT
		R: CCTAAAACTTCAGACACGGA
	TRV1	F: GAAAATATTGCTGCGCCTAACG
		R: ACCTGCCACGGTTCGAAGTA

Table S6. Standard substances used in study

Chemicals	Information
α-Carotene	CAS#: 7488-99-5, BOC Sciences. USA, Catalog#: 7488-99-5
antheraxanthin	CAS#: 640-03-9, BOC Sciences. USA, Catalog#: 640-03-9
lycopene	CAS#: 502-65-8, RHAWN. China, Catalog#: R007285
Zeaxanthin	CAS#: 144-68-3, Bidepharm. China, Catalog#: BD2134
Violaxanthin	CAS#: 126-29-4, Sigma-Aldrich. China, Catalog#: 52444
β-Carotene	CAS#: 7235-40-7, RHAWN. China, Catalog#: R019713
Lutein	CAS#: 127-40-2, Aladdin. China, Catalog#: X109574
Capsanthin	CAS#: 465-42-9, Extrasynthese. France, Catalog#: ES-0312S
Capsorubin	CAS#: 470-38-2, BOC Sciences. USA

Table S7. Gene ID and conserved domains of pepper DNA methyltransferase genes

Database and accession numbers		Name	ORF length (bp)	DNMT1-RFD Domain		BAH Domain		Chromo domain		DNA_methylase domain		Protein size(aa)
				Position	Size(aa)	Position	Size(aa)	Position	Size(aa)	Position	Size(aa)	
pepper genome database	Capana12g000016	CaCMT2-like	3288			206-313	108	467-521	55	354-404	51	1097
										531-882	352	
	Capana01g001654	CaCMT3-like	3489			251-359	109	515-567	52	399-449	51	1164
										579-928	349	
	Capana01g004297	CaCMT4-like	4227			459-561	103	719-774	56	609-659	51	1410
										781-1142	362	
	Capana02g000460	CaDRM5-like1	2820							599-646	48	941
										660-775	116	
	Capana07g000549	CaDRM5-like2	2175							419-467	49	726
										482-596	115	
	Capana10g002486	CaDRM6-like	2244							423-487	65	749
										502-618	117	
	Capana05g002234	CaDRM8-like	2499							573-691	118	834
	Capana04g000012	CaMET1-like1	5580	101-235	135	759-891	133			1117-1161	45	1861
				410-564	155	933-1071	139			1200-1543	344	
	Capana12g001109	CaMET1-like2	5577	101-235	135	758-890	133			1116-1160	45	1860
				410-562	153	932-1071	140			1201-1541	341	

Table S8. Gene ID and conserved domains of pepper DNA demethylases genes

Database and accession numbers		Name	ORF length(bp)	HhH-GPD Domain		EndIII_4Fe-2S Domain		Perm-CXXC Domain		RRM_DME Domain		Protein size(aa)
				Position	Size(aa)	Position	Size(aa)	Position	Size(aa)	Position	Size(aa)	
pepper genome database	Capana09g002341	CaDML1-like	6579	1300-1423		1461-1477	17	1682-1713	32	1716-1816	101	2194
	Capana10g001947	CaDML2-like	6528	1280-1405				1663-1694	31	1697-1797	101	2177
	Capana12g002335	CaDML3-like	6885	1351-1474				1752-1783	32	1786-1886	101	2296
	Capana03g000092	CaDML4-like	5496	992-1109		1148-1164				1423-1523	101	1833

Table S9. ABA and IAA levels in the premature-ripe and green pericarp of the *CaMET1-like1*-silenced fruits.

Sample_ID	ABA (ng/g FW)	IAA (ng/g FW)
V-MG	34.48 ± 5.36	1.41 ± 0.19
V-R	82.10 ± 8.33 **	n.d.

"V-MG" and "V-R" indicate green and premature-ripe pericarp of the *CaMET1-like1*-silenced fruits, respectively.
"**" indicates the p-value of Student's *t*-test was less than 0.01.

Fig. S1-S5

CaCNR (Capana02g001917)

TCTTCTTTGCAAGGTTATTACATCACAAATTCACAGTTTCTCGCATCGCACATAATCAAGTTATTCGGTGAATAGTGTTAACTGTTTAT

CTACTAATAATAAAAAGATTTGAGGATGTTAAGGAAGATGATAAGAAAACAAGAGGAGCTAGCAACTTTAAATAAAGAAATCTCAG

GAAGAGAACTTCTTTATCGATGATCATCAATCGTGATACTCATAACCTCACTTCTTTTTCAGCCAAGTCCTAATCTTGTTTTGTTTCTT

CCAATAATCAAGTCTCCTTGTCAGTCATCTTGTTACTTTCCCTCGAAAGATTGTCGAACTTCACGATAATTGATCGATTCTACCCAAA

AGAAATCACCTCTCTGCTGAAATTTGAATATGAGATTGCATTGTCCTCAACAGCTTCATTGACCATTAGGTCACACCCTTGGACGCG

GCACGAGGAATATTCAAATGAGCTAACTTTAAGAATCTCTACACAAACCTAACATTCTCGTTATACTGCTCAACCATTAAGCTATAAAACAACAC

TACCACTTCAAGAATTACCGACATAACTATGAGAACTGGAAATCTAAGTGGAGACTAATAATTACTCATCAATTGCACTCAGGCTGTAGCTTCA

ATAATTTAATTTGAAAATTAATAATTAAATAATTCATATGGCTAATAGCCACAGAGCTGTCCTAGTGGCTAGTACAGTGCATTATCAATTTAATACT

AGGTCCTATAGCTATGATAGATGTAGCATCACAAAAAAGCTAGGTGCCCACTTATGGATATTAAAACTAAACCAAACCTCAAGGATTATTTATTT

AAAAAAGAAAAAAGCAGCAGCAATTATAATCTGAAACCATTTAACGAATTAAGTCCACATCACAAAAATTAGACAGAAAATTAGCACACGGCAA

*ACACATAAAGGGCTAGTTCATTG*ATTCATTGAACCATGCATTTCTTTACCGTTCACTTCCAATCAAAAATACTACTACTACAAGATTTTA

CTTGCTTGAAAAAGACTACCAAATAGCGATACTTGCAGGAATTTGTTAATTTATTTTGAGTAATGTAAGTGTGCAAGATAATGATGGC

TAGTGTTTTTATGGGTAACTAAGATAATGATGGCTTTAGGTAGACGAGTTAGATATCTAGTCTAGTGATAATGGAAAGCCAGTGCAAC

AATTGAGCACTCAAGAAGTGCAACAAACGCTCTGCTGGACCGACAACGACAACTGAGAGACCAACTTGCAGTATGAATAATGGA

AAAAAAAACAAAAAAAAACAGTAACTTACTTGGCTCCGTTCAGATATGAAGAACTACCCTAAACTATGTTAAATGACTATTACAGGT

ATTTAACTTCGAAATTTTCAGAAAAAGGAATAGCTGCAACCAATTACTAATACATACCCTTGTCGCAATGTCTTAACAAATGAGCCC
ATGTAACGTAGTTCATGAATACTCGTATTAAATGCTTTTGGAGAATGGTTGTGTTTGCCTAGGGTGGAAGAAGGAACTCGTTAACTC
GGGTCCGGGAGAACAGGGCAAAAGAGTAATTGTGCCACCGTTCAATCGACACCAACTCTTTCGATGCCTTCAAAGCCTGAGGAAA
AGAGAGAGCGTAGTCACTACAAGCCACTGGCTTTGGTCTATGGTTGACACAACTCCTCAATTAATTGTCAAAATATCTTGTGAACAC
CGACTAGCAAGCACCAAATTGGATATTCTACTTGTGAACACCAACTAGCAAGCACCAAATTGAATATTCTATCAGCTTCTCCACATC
CTAAAACAGTGAATACTGATTGCTGAGAGGCTTGCTGACTGCCCAATAAAAACCTTGACAAAAGTTAAGTGGGTCCACTAACTAGG
AATGAAATCAACGGTAGATATTCAAACGAACTTTAGCAAAGTCCATCATAAATGCTCTTCACATCTCATGTACTATGTTATGGAATGG
TCACATGTTTCTTTACATAAAAGCTATATCATGTTAAAAGTGG

CaNOR (Capana07g002220)

GCCATAGGTTATGAGAATATTATCTAGAGTCATGAAATTATGATTCCGTTAATTTATTAGATTTGAAAATTTGAGATTCTCTGATTTTAC
TTTTTTTTCCGCCTTATATTACTTGACATATTTGTCAAAAGAAAAAAAAATTATTCCAAAATAGTTGATATTTTCAAAAATCAAGAAAT
AATTGAACACCTGTTTTTAACTTTATCTTTAGAATTTAACGAAAGTAATTAATTTAAATAGATAGTGACAATATTTAATGAAAATTTAA
TAGGGTCATATTAGTCAAGTTACAACCCTTATAAATTTCTCCTTAAAAAATATGTAAAAGCAACATGTCAAGTAATATAAGATGAAGG
AATAATAATTTAGACCAGCTTAATTCAATTGATTAAACCTGAGTTGATATGCAGTTGAGATTGATATATGAGAAATTTTATTTAAATATA
TTAAAATATATTTTTATTTAATTTGTTATGTATAGTGACAATATAAGAGAGAATTTTATTAGGAATGCAAATGAATTGTAGAAAAAAAA
ATTTGAACCTAGTAAGAATTGGTGTAGTTTAGCTCAATCCTTTTAATCCAAGTAAATTGATTCGTCCATCTATTAATTCATTGGACACA

CTTAAGACACCTTTTGACCAACCTACCTTATTTGACATCCCAAATATTTCCCCTAACAAGTTGTGGCTATTCTTGATTAAAAAACGAT
GTTGATATTATAAAAATTATTTACATAATAAGATAACTAGTAAGTTAATTGTGACATTACTATTTCTACTAGCTAGAAAAAGGGGTTAG
TAAACTATTATGACCGATTAGACTATTGATAATTATGGGTGGAGCTAGGGGACACACAAAGATGATTGTAGTTTTCTAGCATTGGATT
TATTTGAAATTAGTATTTTGATGCAATAATCCATAAATTTATGTGTAAAAAATTATTAAAATTTTAACAAATAATACATCTAATCTTACA
ATATTAATGCTATAATGAGTTTAATTATATTTAAATAGTGTAATT*TTTCGTCAAATAAGGTTCAGATGAATTTTCAACTCTAGCTGGCTCTGC*
TTCGTTCTTTTCTTTTTTGGTTTTCTATTGGATATTTGGTGTTCACATTGAAACTTTGACTAATTTAGATCGCTCGTTGCAGGACCTGTTTTGGG
*GGTAGCGCCCTAACAGAATTTTTTTCATACCCAGAACTCGAACCCGATACCTCATGTTAAAGATGGAGCA*GACCCACCACTACACCACAAC
TCATATTGATAGTTCCTTCTATTAACAACAATAATATACACAATATA*ATCGTATAAGTTGAGTCTCGACACATATAACTTTAGTTTTATATTTA*
AAAAAGTATAAATAACATAAATATCAACTCTTTCGGAAGTAATTACACTCTCTCTCACTTTTTGAATTACTTTATTCTCTCCCTATTAATATACATAA
*CATAGCCGACATATACATAGATTCTGTGTATATGTTGGGAATTTTGTAATATGTTCAGAGAGTTGAGATTTTTTG*TAATATTGAAAATATAAGT
TGTGTATTTGTGTAATTTCTAGTTTTTTTTTTTTTAAATGGAGCGGGTAGATACTCTAATTGGTTAAATTTTACGTACTTATTAATAGT
AGTGGCTAGAAAAATTATTTTACAGTATATACTATAGGGATACCAGTGTATAAAATAATTATAAAATGAAATATGCCCCCATATATACG
ATATAAAAAGAAGTTTGGCTAGCCAGCCAAATCTTATTAACCAAACCAAATTTCCAGCTGATTTTCTGTATTCTTGTACTACTAACTC
ACAACACACACACCATTTCTCTCTCTCTGCTAAACATCCAAATATCATAACCACAATTTTCGCACCATACAATTTACTCTTCTTCTTCT
TGTTATTATTATTATTATAATTATATTATATAGTTTATAATCAGCTGGCCCTGATCAAATTAAAAAAAAAAAAAAGATTTTTTTTCGACG
ATTTTATCTG

CaRIN(Capana11g002005)

TTTTTATAATAATTTAAGTTAATATAATAATAACTGAATTCACAAGTTAAATAGTTATAAAGATTTGTGAATTTTTAATATACAAATATAA
AGGCTAAACAAAAGATAATAACTAACTTTACGTGCTCGCATTCTGTAAAGACTAGAAATTCACATCTCTGTATATGTCAATTTTGAAA
TACTGATAGCGTAAAAGAATATATTTATAATTTTAAATAGAACATCTAAAAATTAGGTTAAGGAAGACACATATGAAGACGAGTCGA
GGCTCTCAACTTTTGAACTTACTAATTTTAAATTTATTATGAATAGAGAGTTCTTATTATTTATCTGATCATTTAGAAATTGAAATTGAA
TATCTCGATTAATTTGAATTTGCATTATAAAGATAACTATTAGGTTATGAAAGACACGTATGAAGAGGAGTCGAAGTTTACAAATTTA
AAACGCATTAATTTTAAATTATTCTAAATAGAGAGTTCTTATTATTTATCTAACCATTGAGAAATTGAAATTGAATATCTTGATTAATTT
AAATTTGCATCACGTATGAAGAGGAGTCAAAGCTTACAAATTTAAAACTCCCTAATTTTAAATTATTCTGAATTGAGAGTTCTTATTA
TTTATCTAACCATTGAGAATTTGAAAATGAATATCTCGATTAATCTAAATTTACGTTAAATAGATCACTGAAAAAGTAAAACGCTCAT
TATCAAAAGTTTTTTTTTTAAAAAAAAAAAAAAAGTTTCTAATTAGGTCAATTGTCGTTTTCTTAGAGGGGGGGACTCCACA*CTAAAT
GCTTGAAACGTGGAATTTGACTTGACATCCAATTCTATGAAAAAGAAACATAATTCCTACTTATTAATTTCTACTTTTTCCTTTTTCTTTGGAGGT
TATTCTCTTTTTCTTTTGGACTTTTATTTGGGGATTAATTCAACATAATTGTTTGATAGGTATCAACTAAGATGAGGGTCTATTAAAAATAATTTTT
ATATCTTTGTAAGGTAATGGTAAGATCTACGTACATTC*TATTCTACCTTTTCAAATCCTACTCGTAGAATTATGTTGAGATGTTATTGTTATT
GTAATTCAACATGATATCACAACTCTGTCTACCTCCAACTCAAAGATTGTAAATTCGAATCATCAAAAAACAAAAAGTTGTGGGAG
CTTTTGAAAAGGGTAAAAAATGATTAATTAATTGAGGATTTTTTGATTCATTAAACGAAATCAATAGCTTCTTAATTATTGGTTAAAA
ATAAAGTTGTAGTCCCTGTTTCCTATTAGTTGATCATTTTACTAAA*AATGGTTGTCCCACAATAGTTGACTATCCTACTAAATTAAGAAAAC
AATAATTAATTTTATTTCATATTACCCTGAAATTAATTCTTTTTTAAAGTGTTCACACTTGATTGTAAAGTTTCTAAAAAAAATTTAAGGGGTAAATT*

AGTAAAATTACTTTCTTATTTATGATTTTTTTGAACATGTGTGTGAAAAGAAAAACAACCAACTAATATGAAATAGAGAAAATATTAATGAGTGA

*AATAATATTAGAGGG*AAAAAAATAAGAGAAGATTTTTAGAATGGTGTGGTTGGTATGAATGAAATGTTTTCGAGGAAAATAAGTGTCT

TTTATTTATTTTTATGTGTATGATACGTAAGTAAGAAATATCATTTTAAAGATATTTATATATAATCAAGACACGATGGAATGTTCGAGT

GGGATATATAGAGGCCGTTATTGTGAGGTGGTAGGGATAGGGATAGGGCTAGGGGGTTAGGGTGAAAATGAGGTGCGTGGAAAAG

ATATGATCAACATGAAACATTATTTATAAAATTTATTTTTTCTATTTTTACTAGGCGAATTCTTTTCTTTTTTTTTTTAAGAAATTTATTT

TTCTATTAAAATTATATTTCAAAAAATTTAACTAACAAAACATGATAAATTAAAAAAATGCTTTCCTACACATGCTCATCACTTACAA

ATTTTAAAGGAAAAAACTTTAGTTACAAAGTTAAATTTTACCATTATGTAAGGACCTTACAACTAAAGATTTTGAAAGAGAAGTAAC

CACAAATTTTTCAAATGAAAAAAAAAAAAACAAAGGAACAATCTTTTTCTACCGTAAGGGGGATGTGATCTGTTTGATAGAATTCAT

TCAACCTAGTGGGGGGTTAATTTTTTTTTATATATATATAAATCGATTCAAACTATAAAAAGCTAAGTACACCAAAAAAAAA

CaCYP707A1 (Capana04g001637)

CATCTATGGAATTTTGTCCTAGAATTAATTAATTCATTATCATCTATGTTTTTTCTTTCTTTTTCAATGTTGCCAAATTGTCACTGTTCA

AGTTAGTTAATTAACTAACTAACATGAAGTGCCAAAGGGAGAAATAT*CTTCTTTTCATAGGGAAGTAATTGCAATATATATGTATATTCTTT*

ATTTACATTGCTAAAATCAAATGCTTTATAGTATAAAGTTAAGTGTTTCGACTCCCTTTATGATAAATTCACCTAGTCTTTTATTCATTCTCATTAA

ATAATTCGAAGAAGATATGTAGAGCGATCAAATTAATAGTTTATATCGTAATTATAATGATTTCGAAATTTGAGTATCGACTATTCAAGAACGAGA

*ATCATTTTGCAAGAAAAAGATTACAAAAAAATATACATGAGACTCAATAGAGGTCAGTAG*TAGTCATATCCATTAACACTATTAAAAATACTG

AGGGCCAAAGTCGATGGACAACGTTGATTTTTTGATCAAAACTGATAGAAAATTGACGCATCTACT*TTCGTCGAATTTGGGTTTATCG*

CTTTCGGCAATCTGATGGACAGCATCGGTTAAAACCAATCCATCAATTTTTTAAAATCGACAAAAACTGATGGATAGGATCAATTTTTCAAAAA
*TTTCAAAAATATTGATAAAAATAGCTACTTGGATTTGAACTCGACAAT*TTTACCACTCAATAACACATGCTCTTTGATGAAATGTTTTCCAT
CATTTTATTTATACACTTCAACTATAATTTTGCACTTTAAAAAACAATAGAATCTGTCGATTTTTGAAGAAAAAAAAAAACAGAATGTG
TTGGTTTTATTTTAATAAACTTTGTGGCAAAAAAAAATTATTTTTAGTAGTACTTATAACAAACAAGTAAATTGTAATCTAAAGTGATTA
GTGTATTAATATGTTTGGAAGCACTTACCTTTTATTTCATTTGGATGCCAAGTCATGTAATCTAAAGGCTAATTTGCTTTTAACTGCTT
ACTTTATTTTTCATTTCAATTATGGATACTAATTAATGTATTCCATTATGCTCCATAACAAATGCAAATGACAATTATGTGAAATGAATT
GTGCCAATATAATTAATATTTATCGATTTTAAATTTTAAGTTTTAAAAAATCATATTCTCGGTGAAAATAGTCATATTAGCACCTGATAT
TTACACTCACTTATCCACAAGCAAAAGCTAAATAGCTAATCCCCTTCTCTCATTTTGACCATATCCAAAAATAAAATACAATAAAAGA
ACGAAAGTCAAGAATTCTATCCATTTATCCATGATAAAGATTTATTCACACGCAATATTTACACTCACTTATCCTCAAGCTAATTCCCT
CCTCTCATTTTGACCATATCCGAAAATAAAGAACTCTTATTCCTGGATAATTCCAATGGCAAATTGGCTAAATTATATATTTAACTTAC
CTTTATTTGCGAGGATAATTATAAGTGTTGTACATGTTTGTAACTATCTACATTTTATTTCATTTGGACAAGTACTAACTGTAATTTAAT
TATCTAATGTAAGTGTAACATTGTAACAACTTACTATAAATTTCATTTTAATTATGTGTAATTTTGGTAATTAATGTTAGTTTAAGTGCC
AATATTATAATAATAATAATAATAATAATAATAATAATAATAAACAAAAGCAATATGCTTGTTAAAAAAAATATTACATAATCCCTCT
AAGCGATCGAATAATATTTATTTACACTCGATATTTATGTCCAAATCCTAAAAATAAAGACAAATCAGGACTTATCTCAAATTGTAGTA
CTAGTATATTCACCTATTCACACATCTCTATCCGCCCCTTTCATGCCTATATAAAGACTCCCATCTCCATTCTTCAACAACAACAATAA
CAACAACAACAAAGAAGAAAGATCACTTCATTCAAATTATTATTTTGCTACAACAACAAAAGTTGTTCATTTATCCTACCTATATAAC
AAAAAGAAC

CaNCED1(Capana00g003114)

TCAAAGATTCAAATCTTAAAAATAGTCTCTAGCATAAATACACAATAAGATTGCGTATAATAGCAGTGAAAATGATCTAACACTCTAA
TGTTGACACCTCCCCCTCCTCCAACCCCACCCCTACCAACCCCAAGTTTTGACAGCATAAATGCAGAATCAGATTGCGTAGAATA
GATCTTTTTAGTTCGATCTTTCTCTGAACTCCATATATAAATTCTTTTTACATACAATGAAATCTATCGGTAACTCCTCTTTAATGACTT
TCTTTGAGACAAATGAAGTTGTTACAAAAAAGTTTGATTGTATATATACATTCATTCAAATTTTTATCATTTACAATTATTCTATTATAG
ATAAGTGATAGTGTATTTTCTATATTTGTGATTTTTTAAATTGAATAGTGTATTTTCTATATTTGTGATTTTTTAAATTGACTTAATTAATA
ATTTAATATAAACATCAAATACAAATGATATTTCTTCGTTGAATTCAAAAAAAAAAATATTTATGATTTTATAATTTAATTGTACCGTTTG
ATGTAATTATCGAAAGCAAATTAATGATATTTCTTTCATTAAATTCTAAAAGGAAATAGAATATTGAACACAACATAACTTTGTCTAAT
TTAAATCAAAATCAAACTTAACGACCTTATACTTTTTCCATTTTCAACGTCTCTTTTCACATCAATTATT*TATCTTTGACCACGAAGTTG*
GTTCAAGAAAATAATAATGTTAATGACTTTATCTTTTTCGATTACTTCTACTTGTTTTATTTGTTAATCTTTCGGTGTTAAATTTCTTTGATACATCC
CATATTAGCATAAACTTTTGACTTATTTGAAAAGACTTGTACCTTTTTGCAATTTTTTCTAGCATGCATATAGTTATGGATTAGACAAATAGATATA
TACTTGCATTAAACTAACTATTTTAACTTAAACTCTTATGATGTTCAATAGTATACTTACATTATTATTATTTTGGTATATATATACAAAACAGATACA
AATAAGAATTAGAAAATTCACAAAAGAGCATCAAACATTTAAAAAATGGAAACCACAAATATGATGTAGGAGAAACATGTACGTGTCTCAAAGA
*AAATTCAGTAATTAATAGTTTATACTTCGTACGTATACTATAGGATCATTAATTTAAATGATGATAGCTTGACAAGTCCTTTA*TGGCGGAGACAC
TCTTCTATAACGAACGCAATTAGATACCATTAATTATTTGGTCAAAAAAATTTTTATATCAAACTTCGTTTTCTAAGAAAGAGTGGAC
TAACTTGCTAATACTTTAAACCAATACAATATAAATATTAATTTGAATTAAAGGGTGAAGTTTCACAACCTCTATAATCATGAACTCAA

AACCATATATAGTTTAAATTGCAATTATAAATTGTTTTTACTCGATATAATCTTACATTGATAATTGAATATAATTTTTCAAAATTAAAAA
GATAATTTATTATACTAAAACTTTTACCATGGGCAGGATCTGAAGAAAAGCCGGAGCACAGGGTCTATAGCAAGCATTTACAACATA
AGAGCATAGTAATAAGTATGGATTGTTAGGTTGTGTTTATCAGATTTTTCATACAAGCAAAATGTAGGGACCCTTCCCCCTCCACCCC
ACACCCACATGGGTCATTTGTTGTATTATTAGCAGTAATGTTGTACTACTAGTTAAAGGGTTTTTAATCCTCCAAATATGCATTTTTCA
CATATCCAACACGTGGTCTTCTCCTACATGTTAGAAGTGGACCATCATTCCACAGTCTCTCCCCCCCACTCTATATATACAATTCCCC
GTTCTCTCTTCTCTCCCTCACAACCAAACCAAAACTCACCTCAAAAGAAACCCAAACAAAAAGAAAGAAAATAAACTCATCTCCT
CACTTCCCCACTTCTTTTCTCTCTACAAAATTCTCACATTTTCCCAAATAGTACTACTTACTACCATACACAAAAACATCAAAAAAAA
TAGGTAAAC

CaCCS (Capana06g000615)

ATCAACCATTTCTTCATTAATTGAAAATCTTTAGTCAACTGTAGAATTTAAAATTCAACATTTTGGTGTATGCCTGGATCTTTTTCGGA
ATTTCTGTTTACACGATAGAGAAATGGAATAACTAAACCACTGCTTCCCCTGGCACACCACTCTTGGAGCCATATGTTGGGTTAGTT
AGAAAGAAAGGAACACTAAATTTTTCGAAGACGAATGATATTGTACGGCAACTCAACACAAGTGGAGAGGAAATTGCTTATCTTAG
AATAAAACATAAAAAAGAATTTACCTTGTGTTTTAGAATAAAACACAGAAAAGGAGTTATCTTAAAAAAAAAAAAAAGGAAGAAA
GGATCAACTTTGAAAAAATTATGCGTTTTACCATTTAAAATTGAAAAGATAAATATTCTTTTAAAAGAAGTTGTTGAATGGAAAATAT
TGGAAGAATTTCATTTCATTTTACAAAAATAAAGAGTGTAGAGGGTATTTTTGTAAATCAATATTTTTTCTATAAAAAATATATAAGAA
ATATTATTTTAATACATCAAATCAAATACTGTATAAGAAATAATGTTAACATAATTAATGCAAGTATAGCTAATACCAACATTACTAATG

CAAGTATTACTAATACACCATATTCTATATTAATCTTATATACTCTACCAAACGACCCTAAGTGTGTATCT*ATATCCTCCGAGAATTTGGA*
ATTTGCAAATTCCAAGTTTTGTATCTCCCTTTCCCAGAAATTAAGATAATTCTGGTGCTTTTAGCATTAGAAAAGTATTTATTGGGTAGGGAAAT
GTCATGACTTCACAGCATTAAGCATCAAGGGTATAACTTAATGAAATAGTGGTCAATGAATTATATTGAGAATGACGAGGTCTCTGTTCCAACT
*TTGGTAGACTTTGGAAATGCTCGTCT*GGACGCCGCCCATTCTTTCTAGTCTTGGTGCCATTCTATTTGGTCTGAGAATGGCATGATGCC
AAATTCTACCTTTTCACAATGAGCATTCGACCTACTCTTCTTTTTTCGACTCATTTGACCTACTAGGCATTGGCCAACTTGGCTAACC
ACTTG*AGGAACTAGAGTTCGGATTCAATAGAATCTAATAATTTTAATCAAAAGACTTCATGTATATTGAAAAATCTATTTATAACTAACTTTAAATC*
*GGCCTTTACGTATCGACGTAATCAAAATTGTGTCAGCTTGCCACGTGGGGTCTAGTATGAGTTTGAAATTGGT*CATAGGGGCCCCAATTCC
ACTAATACAGCTGCCGTCCATGCACTACAAGACAAATACACCACTATGTTTGTTAGTGCTTGGTAAATGTAAAACAAACTTTTGATG
AGAATCTATTCGTGGCATCGAAGTGCTGCAAATTGGCTTTTACCTCTGCTACTTCAAGCCTCACTGATTTTCACCCCAACTTTCTCAT
TTCCCTTTCAAGGATTTGATTTTCCAGTTGGGCATGTTAAAAACAACAATTTTCCTCAAAACTGTAGAAATGATTTCTCATATTTTAA
TCAGTCAAATTATTTAAACAAGAAGTTGATTTTTTTTTAATTTTTTTTTTTACAAAAAAATTTCAAATGTCAAGTAAGATTTTTCAAAT
TGAAACTGAATAAGCTGCGACTTTAGAAACAAAAAACTAAGATAAGTAAAAATACCAAAAAGAGTGAATCACATCAATTGAATTCT
TCCAACAGTTCGTTTTTTAGTTTCTGTTTTGGGAAGAGGAGTACTACAAGGTAGGACCTCCAACAATCAACAATATCTAAGTTGCAA
AAGTTTTTGTGCGTTTTTTAGTTTCTGTTTCGAGAAGAGGAATACTACAAGTTCGTTTTTTAGTTTCTGTTTTGGGAAGAGGAGTAC
TGCAAGGTAGGACCTCCAACAATTATCAATATCTAAATTGCAAAAATTTCAGTTCGTTTTTAGTTTCTGTTTCGGGAAAAGGAATAC
TACAAGTTCGTTTTTTAGTTTCTATTTTGGGAAGAGGAGTACT

CaPSY1 (Capana04g002519)

TAAGTTCTGATCTATTAGAAACAACTTCTCTATCTCCCAAAATAGGTTGGGGTAAGATCTATGCACACGCTTGACCTATTTACGTGAA
ATAAGCTGGTCAATTGTGAACAACATAACCGATAACAAGTGCGAGCGCAAGGGGTCCAAACCTTTTTGTCGAAAAATTATATAGTAT
ATGTGAGTATTGTGATCACTTTAGTAAAAATATTGGCCACACTATTATTAACAATTTATATGGTTACAACAATTTCTTGATGATAAAAT
GACTTACTTCAAACTTGGTTGAAATTTAACCTAAGAGGACAAAACTTGAATCCTAATCCAAGTCAATTTGGGATCCTTATGTTTGAG
ATATATTCAAAATAGTCCTCCAAATCTAATTTTTTGAGCAATTATGGTCCTTTGAAGGTGAATACTTTTTGTTTTGTTTGTAAATATTTA
AGAAATTCTATCTGTTATCATCTACTTTGAAGAGTTCGATGTTGCTTACTGAGGTGCAACTTCTCCTATTTTTTTAAAAATTATTTTTG
GCATCTAGTGAAGGGAAGTGTTGATGTAAATGATAAAGTTATTGTCATGTCATTAGGAGGTTATGAGCTTAAGTCGTGGAAATATTTT
TCAGAAATGCAACGTAAGACTACGTATAGTAAATTCTTGTGGTTAGATCCTTCTCGAACTCCATATTTAATGGGAGCTTTAGTTCATT
GGACTGCCCTTTTATTTTTTGCCACTAGTGTAGTTTTCTAGGATTTGATGAGACTTTCTCGATTTCTCGAATTAGAACCTTTTTCACAG
TTTTCGGCAAACTTAATAGATAAAATTTGTTAAAATATTTGACAGAAATAAAAAGATGTCCACTTTTGTAAATTTAAAGGACTAAAA
CTGCTCAAAAATACATTTAAGAAACTAATTTAAACCTAGTACCAAACATAAGGGACTATTTTGTCAATAATTTTGAAAATTTACGTGT
CTTTATTGTAACCAAAGAGTTCATAGCAACTTAAAAAAGGGGTAAAATGAGCTGATTTATTCTGAATCACTCAAAATTCCAATAAAA
TTATACAAAGAAAAATGAAAAAAAAATTACGTAATATAATAATTTTTAAAAATTAAATAATGAATGCTATAAATAGACCAAATTATGTG
GAGCAATAAAAGAGGAGAAAAACCAACAAACTACTTGTAATTCTTGAAGAATACAAAGCAGACATTGTTGAAAAGTGGCCATTTT
TGCA*AGGTTTGTTTCACTTCAATTCTTGTTATCGAATTCATAACTTTAGTTCAAACCATGTAACATGTTAACTTAAAAATATTATAAAATTTAGAAT*
TCATAAACTTTAAATTTTGACTCGTTATGTTTATTGATATACAAAGTTGCAATATTTAACATTCTTTGTAACTTTCTATTGCTATTGTAGTGTTCTTG

TTGCTATTCTAGGGGCGGAGCTAGAGTGTCAGTTACGCATTCAGTTGAATTCAGTAACTTTAGTTCAAACCCCGTGATGTGTTAACTTAGAAA
*TATTAGAGTTCAGAATTCATGAACATCA*AATTTTGACACGTTACATTTCTTGATATACAA*AGTTGCAGTCTTTGACAATCTGTGTGGCTTTT*
TATTGCTATTGTAGTGTTCTTGTTGCTATACATGGGCGGAGCTAGAGTGTTAGTTACGGGTTTAGTTGAATTCAGTAACTTTAGTTCAGACCCC
GTATCGTGTTAACTTAAAAAATATTAGAATTCTGAACCCATAAAGTTCAAATTTTGACTCGATTCGACTTCTTGATGTATAAAGTTTCAATCTTTA
*ACATTCTTTGTAACTTTCTATTGTTCTGGTAGTGTGTG*TGTTGGTCTACTGGGCGGAGCTAGAGTATCAGTTACGGATTTAGTTGAATTTA
GTAGCTTAGCTTAAACCCTGTAACATAGTAACTTAAAAATATTGTAAAATTTAGAACTCATAAACTTCAAATTTTGACTCATTACATTT
CTTGATATACAAAGTTGCAATCTTTGACATTCTGTGTGGCTTTTTATTGCTATGGTTGTGCGCTTGTTGCTATACATGGGCGGATCTAG
AGTGTTAGTTACGGGTTTAGTTGAATTCAGTAACTTTAGTTCAAACCCCGTAATGTGTTTACTTAGAAATATTAGAATTTAGAACTCA
TAAATTTCAAATTTTGGTTCGTTACATTTCTTGATATATAAAATTGCAATCTTTGACATTCTCTGTGGCTCTTTATTGCTATGGTAGTGT
GCTTGTTGCTATACTAGGGGTGGAGCTAGAGTGTCAATTATGGGTTTGGTTGAATTCAGTAACTTTAGTTCAAACCCCATAACGTGT
TAAACTTAGAAATATTAGATTTTAGAACTCATAAACTTCAAATTTTGACTCGTTACATTTTCTTGATATACGAAGTTGCAATCTTTGAC
ATTCTGTGTAACTTTCTATTGCTATTGTAGTGTTGTTGTTGCTATACTAGGGGCAGAGCTACAGTGTGAGTTATGTATTCAGTTGAATT
CAGTAGCTTTGGTTCAAACCCCATAACATAGTAACTTAAAAATGTTAAAAATTTAGAACTCATAAACTTCAAATGTTGACTCGTTAC
ATTTCTTGATATACAAAGTTGCAATCTTTGACATTCTTTGCGGCTTTTTATTGCTATGGTTGTGTGCTTGTTGCTATACATGGGCGGAG
CTAGAGTGTTAGTTACGGGTTTAGTTGAATTTAGTAGGTTTAGTTCAGACCCCGTAATTTGTAACTTAAAAAACATTAGAATTCTGAA
CCCATAAAGTTCAAATTTTGACTCGGTTCGACTTCTAGATGTATAAAGTTTCAATCTTTAACATTCTTTGTAACTTTTTATTGCTATAG
TAATGTTCTTTTTGCTATACTAGGGGCGGAACTAGAGTGTAAGTCGAATTCAGTAACTTTAGTTCAAACCATGTAACCTAGTATCTTA

AAAATATTAAAATTCGGAACCCCATAAACTTCAAATTTTAGCTCCGACTATACAAGAATAGAATTTGAGAGTGGCCTAAAATTTAGT
GTCCATTGTTTAGTGGAGAACCAATTATCAAGGTTTGTTTCCCTTCACTTCTTGATATACAAGTTGCAATGCTTAACATTCCCTGTAA
CTTTCTATTCCACTGGTAGTGTGCTTGTTGCTATACCGGGGCGGAGCTAGAGTGTTAGTTACGGGTTTGGTCGACTTTAGTAGGGCA
GCCCGGTCACTACAGCTCCCGCTATGCGCAGGGTGGCGGGAAGGGGCGGACCACAAGAGTCTTTTAACATTTTTGCAAGGAGCTG
TTTCCACGCTTGAACCGGGGACCTCCTAGTCACTAGAATTCAGTAGCTTTAGTTCAGAATCCACAAACTTCAAATTCTAGCTCCGCC
TACTAGTGACTATAAAATAATAGAAAATGAGCACTTGCCTTATGAATATAGCTTCTACGTGTACCAAAATTAGAAAGTGAGGTGCTTA
TTATAATCTAGTTGACTAAATATAGAAAGATCCCATTACCTCCAGAAAGTGTGATTCCACTTTGTGCTTTCAATAGTGTAAAATAGTTT
CTCAAACATCCTTTCTTTGTGCCATTGGTAGGTAAGATTGCTGTTTTGTCTTGTTTTGGAGATGTTTTTTAAAGTTAAAATTGTTTATA
CTCAGGAAGTCTATAATGCTGGTTACGAGTTCATGTGAACTCAGTAGTTTTCGCTAAGCAATCCACTAATATCTATAAATATTTGACC
GTGAACTCCGCTTGTATCATTTCATTAACTTGAGGTCGCTATAGGAGCCGATAAACTTCCAACTTCTGAATCAGTTTTTGTTTATACT
CAAGTATGATGTTTGGTTTATCTCATATTGCAGAAGCCAAGAAATAGGTTATTTCTTTGTTTGATAGTGGAAGTATACTCTAGTGGGA
ATCTACTAGGAGTTACTTATTTTCTATAAAGAAGACAAAAACCTTGGAGTTGCTTTAGACAACCAAGGTTTTTCTTGTTCAGAGGAA
TATAATGTAAAGTATAGTAGACAATTTATTTTATCGTATACATATTAATAATTATTTTCACGATTCGAATATATATAACCGATAGATCACA
CAATAATAAATATTAGTGTTGCTCATCGAAAACTCCGATGCACTAATGTTTGCCACTAATTCTTAAGATAGATAACAAACACATCTAA
ACATTATTAATTAAGTGTATATATACAACATATTTTAAACTTATTCTATAACTGGATTTCAATTTAAAAAAAATAATGATGTGTCATGTC
CCAAAGTTAGTTGCACTCTAAAAAAAGTTAAAAGGTTTTTAACCAAAAATAACTTCTTGACTATAACAAATTAGAGTTGGAATTAAT
AATCAAAACATATAAAAATTGATATTTTTAAACAAGTTTTACACCATAATGTAGCAATCCATCCTGTTAGTGATATTGTCTGCTTTAAA

TCTAGGAATGTACGTCTTTAAAATGCGTCATTAGTGGGTAAGACATGCTTACTTAAAACACGTCATTAATGAATAAGATTTGTTTACT
TATATACTCAACATCTCTCATATATTTTACTGATGTGAAATTAGTTATCTTAAACCGGAATGTCAGTACACTTCATTTGTATCTTTTTTTA
TATGAGCCATTATCATTTACATGTAAAAGTGCACCTTAAAGCTGGTTAAGCTTATAAACTATAAATTGTTCATTTTTTCTCGTTTAATA
ATCAATATCTACTTAACAAGGCCTGTTTAATAGATGATAATAGTTTAAGTAGAAAAATGAAATTGTAACTTTTTTACGACTTTTAACAT
TTCAACTATCAGTTAGTAATATGCTCATCCATTACATATTTTAAAGAGAACAAAGAACCATTAAAAGGTTAAAAACTTATTATAAAGT
TAAATATTTTTTCAGTATATATGAAAGGACCTTACAAGTTACAACTAAATCTTTTGAAAGAAAAGTATCGGTCACTACTAAGTTTTCC
AAGAAAAACAACAACAAAGGAACAATCTTTTTCTAC

CaPDS (Capana03g000054)

AGTGAGCTTGTGTGTTCAAATAAGACTTTCCTTTGTAGAATATGTGGCGGTATTATATGGCATTAAAGAGATGGATACTAAACAGGTC
ATTATCCTTCTCAGTAATTCACCCTTTAGTTTTCAAGTCAAGAATTCAAAGCCAATCCAATTTGACAAACCTA*AGTTACTTACACATTT*
GATTGTTCGATCAAGAATATCCATATCATATAACCAATACATATATGACAATTATTTTTTTAATAGAGAAATGATTATTTATGTTAATTTTCATTTTTTT
GTTTTCTGTCTAAAATAATCATTTAATTATTCTTTTTTCACCCGAGATAAAAAATAAAAATATTAGCTGTACATTTATTTAGTTAAAAAGAATTTGGT
*GTAATTCGTTCGTGACAACATACTAGTTAGTATAAGGGATAAAATTAATGCGTGTGGTATCT*CATTTCCTCCTGAGAATTTACAAGACGGTT
AAATGTTAGGTACAAACTTAAGACATAAAAAGAAGAGAAACTTTTGATCTCCCAAGTGTGGGTGTGGTTATGGTGGGAGGGACGT
GTCAAATTTTGGAGCGTAGCCAAACAGTCGTTCCAGTTACAGCAGATTGAATTAAGAAGATTACATATTTCCAGCTTATACCTAATA
ATATTATCCCATCTACGGAAGTGGCCAAACCACCAAAAACAGGCATTTCCACCCAAATACCAGAAAGATCCAACAAATATAATACTT

ACTCCCTTTCTCCCAAATTACTCTTCATAAATTCACATGATACATTAATTAAGAAAAATAATCATAAATTGATATGATACATTAATTTAA
AAAAAATAATTAATAATATGATTAATTTATTATAATATATCTATTAAATGATATTTATATTTTAATTTGAAGAAAAATTAATTAATATAAAG
GATAAAACCTGATTTTTTTTTTGTATTAATTAAAAAAGATAAGTAAAATGATAAATTAAATTAGAAAAATTGATTAATAAAAAAAGAC
AAGTAAAATGAAAAATGAAAATAGAAAATTTGATACAGGTAATTTGAGGCGATGTAGTATTAAACAACATTTAATTAAGCCTTCCTA

CaPG (Capana10g002229)

ACTCATTTAATTATATTTTTATTCTTAATTAGATAATAATAATAACTTTAGTTTTAAATACAATAATTTTTAAGTATCTTGTATAATATTTT
TGTCCTAGATCCTCATGCATTTTATAATTATTTTCTTCGTAAAGTCGTTGGTTTGATAAGGTTCTACCCATTAATGTAAACTATAAATTA
ACATTGTTTTTTTTTTTGAAAGATCGCTAACATCGTTGCTACTCCATTCATATTTGCTACTTAACTAATTATAAAATAAAAATAAATTTG
TTTTAGTAATAATGTATTTATACTTGCTTTTACTTGTAATTTATGTTGATTTTTATACATGATGGTAATGTATATAACTGATAAAAAAAAC
AAAATTCATGTGGTATAAACAAAAACAAAACAAATAAATCTTCTTCATGTGAAGATCATATGGTATACAAAAAAAACAAAATTCAA
GTTATTCACGACATGACTAGTCTTCAGATAAAAAATTTATTAACAGATTTGCATGCATTTTAAATTTATTTTTATTTATATTTTTGTAAT
TCAAAAAAGTTTTAATTAGGAAAGTTAATGATTTATATAATTTGGCAAAGCTAATTTATACTAATATGATCGAAATATACACAACATTG
TAATTAATTGAAGCAACGGAAAACACCTTAACAAAAAATTTGGCTCATGCATTTATTACGAAATGTAATTTTTGGTTAAAAGAAACT
AATTAGTGATGATTTTTTTTTTTTTGGTTGTAGCTAATAATCTCATGGTTAGTTGATGTTGCTAACTGGTTATATCAGGTTAGGGAAAA
AGCATCGTTTCCATCCTAAACTATACCTGAAAAGTCTAAGCCACACCTAAATTATACTAGTGACCTATTACATACCTTAACTATAAGA
AAGTGATATTATTACCTCCCCGCGATCCCCATTCTA*AGGCGTGTGTGTTACACTTATTTTAGGCGCATCCAGCCTATTAAATAACGAAAGTA*

AATGGCTAAAAACATTAAGGGAAAAGTCAACGTTTCTACCCCAAACTATAGTTGAAAAGTCAAATCCACACCTAAACTATATAAGTGACCTATTA

*C*ACACCTTAACTATAAAAAAGTGATACTTTTTACCGACGTGGTGCTGACGTGGCAGCGCGTGTAAAACACCACACTACATGCAAGT

GGTGGTAGCCTGATAGGGTGTAAATAGTTTCACTTTCTTATAGTTTAGGTGTGTAATAGGCCACTAGTATAGTTTAGGTGTGGCTTCG

ACTTTTCTGGGGTGGAAACGATGTTTTTTCCCTATCAGGTTATTATTATTATTTTTTGCATATCATCACTACATTAAATATTTAATGATAG

TGACAAATTCAGAATTTTGGGATTATGGGTGTCTCCGAGAGATTCGAACACATATTGATGATCAAAATTTAAAGATTCTGCCCAGGG

TCGGATCTACAGTTTCGGGTGGGAGTTTTCGAGAATCTCTACCTACTATCGTAGATCTCGTAAATATATGAAAAAACAAAAAAAGTC

AATAAAATTTTGTAGATGGAAACTCACACACAAATTTTAGGCTGATTTGATGGCATAGAAAGGACCCTTGAATTAAAATCAAAGCAC

TCAGTTATCTTATTGGCTTCCTGATGCTTTTTGACTATTATACAATTTTTTATATAATTATACTTTATTTGTGTCAAATTTAATGGGTGTT

GAAGCACCACAAAAAAACGCGTAGGTCCGTCCCTATTTAATGAAATTAAAATAGATAACAATCTTAATACATAAATGAAGGTAAATA

GATAGATCCTACCTCCAAATCATATTTTTTTAATGAACGTGAAAAAGAAAAATACGATAAATAATTTAAGACGAGAGCCAATAGAAA

TTGGCAGAATGTCAAAGAAACTCTTTCGTCTAAATCTCTCTCATCCAAATTTAAAATATTACAACTATAGCCATAATTATCTATAATAA

ATTCAAACCCTCTAAAATCTATAAATAGCCAAACATTTTG

CaPME2.2 (Capana00g004152)

TTTGATCTATCGAGATACATAATTAGCTCCTGATATATTCAATGAAGAGATATTTTTCTTTGTAGAGATACATAAGTAGCTCTCGATACA

TTTTTCGATTTTAGGTCTGTCAAGATACATGATACATCCAACGAAAATATTTTTTTTGTTGTTGTAATAGATACGTAATTAGCTTTTG

ATCATTTTTTTTTTCCGATTTTAGGTATGTGGAGATACGTAATTAGCTCACTGATACATTCAGCGAAAATACATAATCAGTTGATATTTT

TATGAGGATAAAGATTCGTGAATATATGATAAGTTAAGATGTATATTTATTTTATTTTAGCTTTTTTTTTTCTTCCCTTCAAAAGATGTAT

AATGAGTGGTTTGATTTTGACAAAATATTTGCTCTTATGGGCCTCCCACTAAAAGATTTAGTGCCATTTGTGTTCTTTCATTGTCTTG

ACAAAGATATTTGTCTAGGTGATAATTAGTGCTCTTTTCTTCTACCAATCACAATTTTTTTATTTATTATTATTATTATTATTATTATTATT

ATTTTATATCAGAAAAATTAAAATAAATTTCTTAATATCATTTCATAAGTACGTTACATCGCTTAAGTTTTCTAAGAGAAAAACTTCAT

TTTCTTGAGCGCTTCTTCCCCTATTATCAAATATTCTTCAAGTTTATGACATTTGTTCGCCAAAAAATTTGCACATTAATTTTCGTATCT

CATACGTGTATGATAATGGTTCTGTTTACGTTCTGTAATTATCTACATTCTGTAATAATGACTGTAAGATGACATTTTTGTCATCTTCGT

TGATAAGCTTTGATATTTTATTTGATACTCCATGGTTTCATGATGAGACTTGGAAGTAGACTTCTTTTCATTTAAATATCTCAATATTTA

CTCAAAATTTTCACACTTTAATATGAAAAATGAAGAGATTCGATCCATCCTAATAATCTCACTTCTAGAAAGTTCACTAACAGAATAT

GTAGATGGTCCATAGGACTATGTTGTTTACCTACTTTGGACAAAGTGGTTGTGCTTTAAATCAATTCTCTCAAATTTATCACATTTTTC

TAATCTACACCATATAATAATGGAGATAT*TTGTGAGTAATGACTTGTTGAATGATCACTAGCCATGCCAAAGTACCCAATTTTCTGGGCCAA*

TATAGTACCCAATCACTATATATTGTATTGTATTGTATTATATAGTAGTATTAAATTCATAAATATAATATTTCAATGACTCCAAGAAAAGTAAAATAT

*AAGGTAAAGTTACTTAAAAATAAAAGAGATAATACTCAATTACCCTGAACTATATCCAAAAAGGCTACGATACACCTTAATTTTAAGGGGATC*CT

ATTATTCCTGAACTAATTAAAAGTATAATTTAACACCGTTAGTGCCTACGTGGCACAACACACTGAAGTGTGCCACGTAGGCACTAA

GGATGTTAAAATTACATTTTTAATTAGTTCAGGAATAATAAGATCCCTTTTAAGTTGTTGAGGTGCGTCGTAACATTTTTGGATATAGT

TCAAAGGAATAATTGGGTATTTTCTCTAAAAGAAAAATATGATGGAGCATAAAATAGATTTATTAAATACTAAATGAAGACAAAATG

AGAGAGATAAAAAAAAAATATAAAGTAACTAAATAATCAAAACCAAATTGATTCCTGAGACTTTTCGTTGTTATCTTATCATAGAGA

TAATCATACAATACAATACAAAAAAAACTTTTAAGTAACAACCAAAACAAACATTACGCTAAATTAGGGATACTTGTTGATTTTTCA

GCAAATAGAGAAATCTGCAGGGACCAGTTTGTACTCTATTATTCCCTCACATATGCACCCAACATATCGAGGACAACATCACTAGCA
AACAATTATCTCAATCACTCTCATCTAGTTGGGAGGTCGATAAGACTGTGGGACCCTTTATCAGACCCTGTACAGAGCGGCATCAGA
CTATTAGCAGAGAATTACTCAAATCCAT

CaCEL1 (Capana01g000166)

GAAAAACTATGAAAAGAGCACGGGTTCATATGCCCCGATGGTTACACGTAAAATCATATCGCCCCTGCATTTCTCCCGCCAAAACCT
TCTCAGTCAAGCTCAAGCTACTAAATATACTCAGCATCGCGATTTACATCTCTTATGTAATTTGCTAGCTATTACACATAAACAGGTTA
TCTAGGGATTTTAATTTTTGAGGGTTCCAAAAAGATTGTAATTAATTATGAGAATCAAAGTGTGACGCTAATGAATATGGGCTCATGA
GAGCCATATAATAGCTTGCGTAATCTATATTGAATTATTATAAATGGATAATGAAGGTGCATATTTGGTCTCCCTCTTTACGTTATCCAT
CTTTATTGCCATATTTTTCAAACTTTGCAACTTCCAAGTAATGATTTTCCTTTCCAACTTTCTCATAAGAGAGACGTTGCATGTAAGTT
ATTGAATAAGGCAAATAAAGGGAAATTACAATCCCTCATGTTAATATGTACCCTAATTTGTATCTAAACGAAAAGTACTTCGTTGCCT
TTTTATCATCATCTTTTATATGTCTTTTAAATATTTAAATTACGGAATACTGTAACTTATATAGTACTTCTTAGTTCATGACCAAAATTGG
TTAATTCGATTTCGATCTCCTTTTGCTTTGGAACAACCGTTAGTATGAAGATAATTTTGAGCTAGTTTTTTTTGGTTTCCCAAGGTAA
CCCCACAGCCGGCAGTCGTGAGACTAATCTTCCGTTTCTATGGTCAGCGCACTAAGCGGTAAGGAACTGGCCACTGAGTTTTTTCC
ATTCATTAGAGGTGGGGATCGAACCCCCAGCCTCTTGCTTAAAAGGTGAGGTACTGAATCACCACACCAACTCTTGTGGTTAATTTT
GAGCTAGTTGACTAGTTGAAGAGCATTTTGAGCCAAAGTACGTAAAGGAGGATAAAGTCGACATATTTAGGATATTTTTGGCCCTTT
ATAACTTGTTTTAACATGTTACATGCCTCATTTTCTTCATATTATTTATTGCACTTACTTAAACAGTGTGAAAGTTATTGATTAATGTGT

<u>ATAATAAATTGAACTCAATTACGCACACGAATCATTCTCACG</u>TATAGTATATAGTTTTCTAACTTTTACACATAATTGGTGTGTTCCCA
AATTTTGAGCTTTGTCAAAAACAAAATGGTAGACACCCAAAATTTGTGCACTCTACGAAAAAAAATTCAATTTATGTTAGAATTTTT
AGAGTTTTAAAATTCGTGCACTCTTCGAGAAGAACCCAAATTCTGTTATAGTTCTTGGAGTTTACTCTAAGGGCAAATGGCATTACA
AAAACTCCGAATATTCATAAAGAGATCAAAAGTCTTTCTTCTTGATAATCAAATACATCAAGAAGATACACAAACACTCCATCTTTTT
TATGGAGATGAAATCCAAATATTGTTACGTTCGAGAAATACGCTACTAACAGTCCTCGAATTACGGAAAAATTCATATCAAATTCAA
ATATTCCTAAGTTTGAGAAATACGCTATTGACGGTCCTCGAATTACGGAAAAATTTATATCAAATTCAAATATTTCTATGTTCGAGAA
ATACGCTACTGACGATCCTAAAATTACGGATAAATTCATATCAAATTCAAATATTTCTATGTTCGAAAAATACGCTACTGACGAACCT
AGAATTATGGAGAAAATCAAGAGAGGGAATCAAGGAAAAAATAAATTTGTAGCCACATCATTTATCAATAAAAAATCATGTTTCTTC
ATATTTTTACGTGTGATTGCAATTTATTATTCATAAAAAATTATTGGAAACAATTATGGCAGGCGTAAAGATGGAAGCTCTATGGGTAC
TTCAAGATTCATCTTAGAGTTTATCAAGTCTACACACCGACAAGCATTGAAGTGGACATTTGTAGACATCGAAAATTTGTGGACTCT
ACAAGAAGAATTCAATTTCTGTTAGAGTTTGTAGAGTTGTAAAATTTGTGACTCTTCAAGAAG

<u>*CaCEL2* (Capana08g002622)</u>

GATACAGACCTTAGTTTAGGGACCTATGTGAATTTTCGGAACAACTTTGAGGTCCTATTGATGGGTTTAACTTTTTACCTTCTATAGT
GTTATACATGCGCATCTAAACCGACAAAACATGCAAGGGTGTTTTGATACTCCATCCATTTCATAATAAATAAATTATTGCATTTTGAC
ACATATATTAAGAAAAAAATATTAAAGACATGAATTTAACACAAGTTTTGATTTTTACTCAAAAAAAGAAAAAGTTGACCTTGCAAT
ACTTTTTCAAAAGTTAATTGGTTGTCAAATCATAAGGACAAATTTTTAAAAAAATTCAATGATTCACTTCTTTTGAAACAGCAATAA

ATACCCCAACAATTCACTTATTCCGAAATGGAGGGAGTAATATTTTTGGTGAAAATAATGAATTAAAATAAATAAATATAATATCCGA
AGTTTAAGTTGAAGTTGAAAAGGGGTGCAGTTTGGTTTTTGGAATAAGATGGGATATTTAGAATTACGTTTATACTTTTAATAAAATA
CAGTATAAATTTAATCTCAAACTTATTTCT<u>TATCCTATCAAAATAGGTGTCATGTTATATCACTGTCTTTAAAATTCTAGGATTATAATTT</u>
<u>CAGTACAATTTAATTCGCAAATCAAATGATCATAGTGTCTGGACATAAATTTCATTAGCGAAGATGGTTTAATTTTTTCTATTTTAAAT</u>
<u>TAAGTATCTTAATTTGACTAAATGAAGTTTACGATACAGTTCATTATCTAATCTTCATCCTCAAACTCCCACTCCCAATCCTTTCCAAC</u>
<u>CTTCACAGTGATGTGTTAGATTGTACATGTTTTTAATTTACTTATCAAATATTAAAAAATATGTAAGACACTTATTTTTTGAAAATTTTT</u>
<u>TCTTTCTTAAAAAACATTTTCCT*AGCACACCCAAAGTAGTGAAAAAATAACAACAATAATATACCTAGTGAAATCCCACAAGTCATGAAGTCT*</u>
<u>*CGGGAGGACGCAGACCTTACCGGCCACTAACTCGGAGAGATAGGGAGGTTGTTTTCGAAAAACAAGTGGGTGGGAATAGTTGGAGTAGCT*</u>
<u>*AGATTAGAAAACAAAAATTAGAGGGACATAAAATAAAAGAAAGAGGTAATGTAAGTCACATTTCTCTTTGCATGTG*AATTTTTAACTGACACT</u>
<u>GCCTTCTTTTGTCTTTCTGTCGGCGCTTATTTGCTTTCACTTTCCTCCTCCTCCAATCTCTGCTCTCTATTTTTCCACTCCCGTGATCCT</u>
<u>TTCCTTTCCTTTTCTTTATTTTCCAACCAATAATACTTTATTCAATTTACAATTAAAAAATTATCTACATTTTTATGCTGATTTTAAAAAG</u>
<u>TAGTATATATTGATTTGGCGCGATTTTTTTTTTTTTTTTACTTGTAAGTTAAAATTTAGTAAGATTTTTAAACACCATGTAGTTATTGGT</u>
<u>GTTTATGGTGTAGTCTAGAAAATAATCAATTCTTAAGTTGAAATTGCAGGTTAGACATAAACAATCGATAATTTCTTTTAATCTGTCTT</u>
<u>AATTTTGATAAATAAAATTACTTGATTCCTATTATGGATACAATTGAGATGCATTCAG</u>GCTTATTCGAATACCATGAGTATTTAAAAGA
AAATTATAATTTGTGTGTATAAAATACCTTTGACACTTGTTATCTTAAATATGTTAAATATTTGTGTGATAAATATTTCTTATTACAAATA
AAATTTAAAATTTATAGTTAAATATTTTAAATATAATAACATGTCATTTTAGAAACACATTATTTCTTATTAAAAATAAAATTTAAAATTT
ATAGTTAAATATTTTAAATATAATAACATATCATTTTAGAAACATACTCCTATTAATTAGTAGAAAATATCAGCGTGTAAACTCACAAGT

TTTTCAATAAAAATAATTAATTTTATATTTTTTTCAATAAAAATTCTAATTTTGTCCCTGTTAACATAATCACAAGTTTTAACTCTACAA
AGTAGTATACACTATACCATAAAATAAA

Figure S1. TSS up-stream regions for McrBC-PCR and bisulfite sequencing. Fragments analyzed with McrBC-PCR are shown in underline. For the bisulfite sequencing, Sequences analyzed with bisulfite sequencing are in italic and marked either by gray (PCR fragment 1) or by dark gray (PCR fragment 2)..

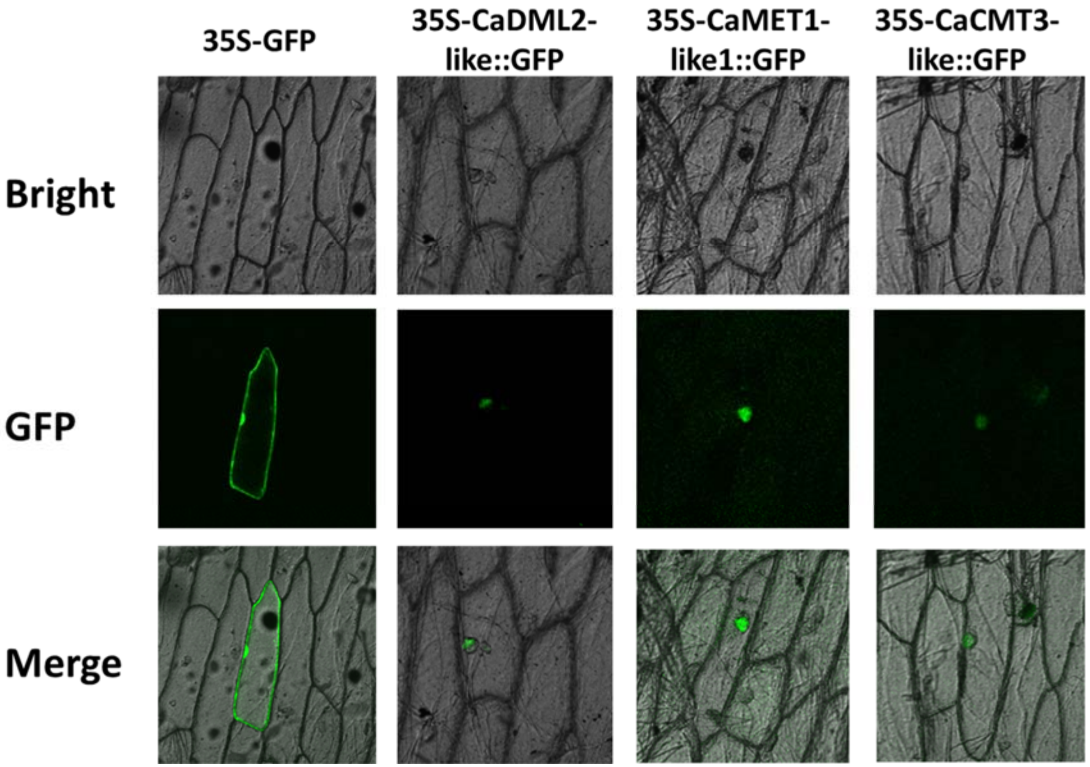

Figure S2. Subcellular localization of CaDML2-like, CaMET1-like1, CaCMT3-like in onion epidermal cells.

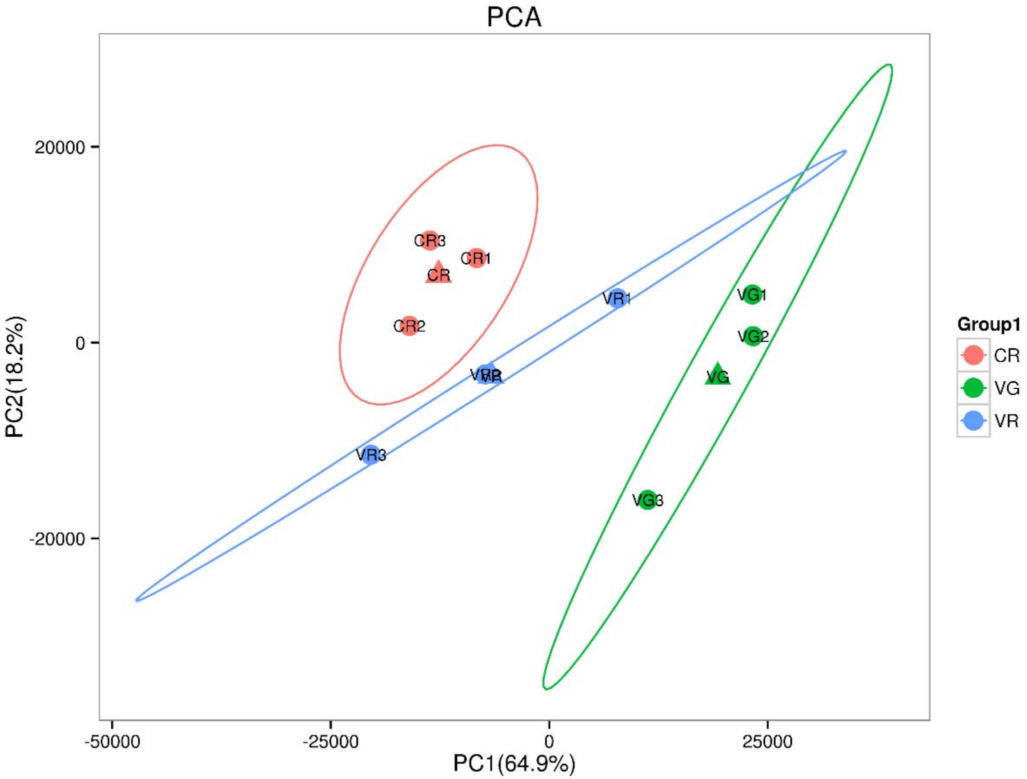

Figure S3. PCA of the RNA-seq samples. CR, VG and VR indicate red-ripe negative control pericarp, green and premature-ripe pericarp of the *CaMET1-like1*-silenced fruit, respectively.

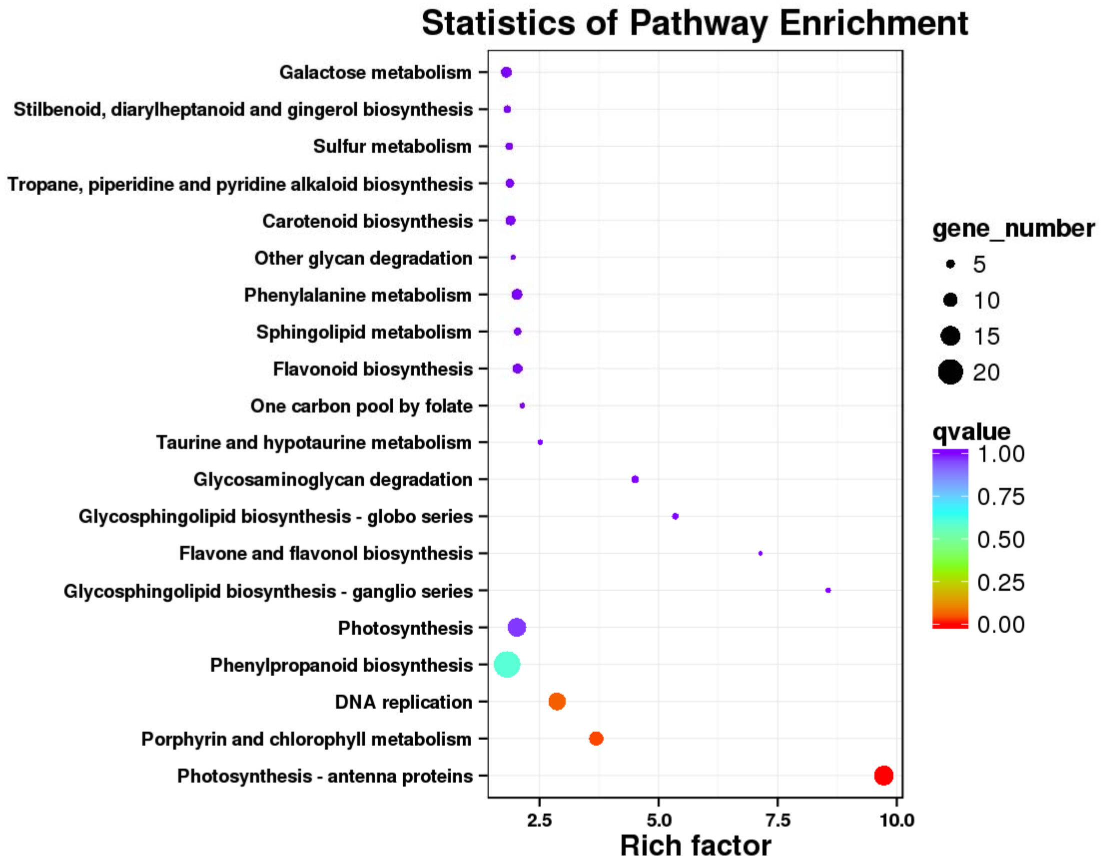

Figure S4. Statistics of pathway enrichment of the DEGs between the premature-ripe pericarp of the *CaMET1-like1*-silenced fruit and the red-ripe pericarp of the negative control fruit.

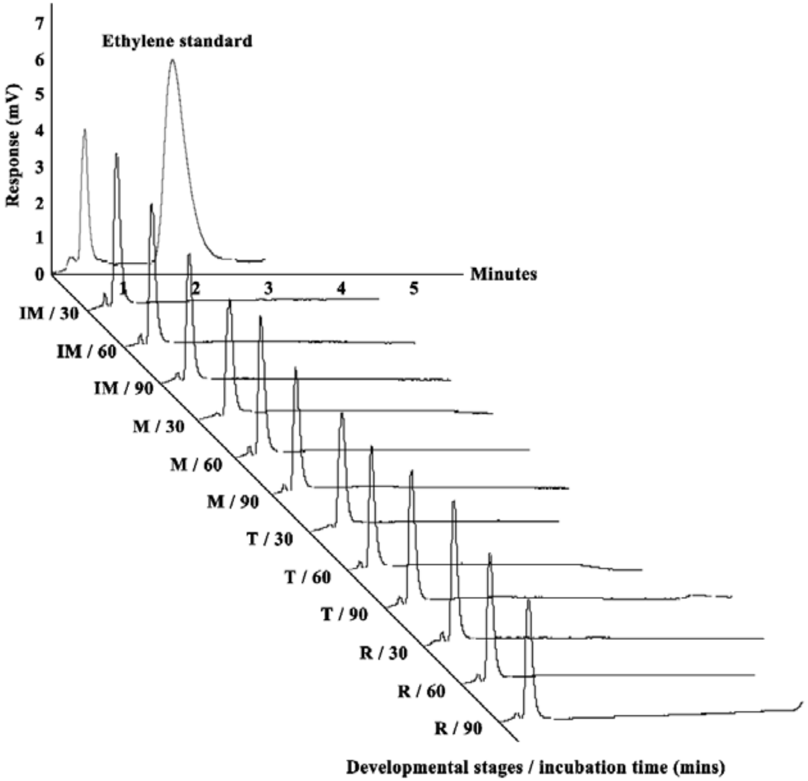

Figure S5. Ethylene production of pepper fruits at different developmental stages. “IM”, “M”, “T” and “R” indicate immature green, mature green, turning and red ripening stages.
